# Criteria for selecting sentinel unit locations in a surveillance system for vector-borne disease: A decision tool

**DOI:** 10.3389/fpubh.2022.1003949

**Published:** 2022-11-10

**Authors:** Camille Guillot, Catherine Bouchard, Cécile Aenishaenslin, Philippe Berthiaume, François Milord, Patrick A. Leighton

**Affiliations:** ^1^Groupe de recherche en épidémiologie des zoonoses et santé publique (GREZOSP), Faculté de médecine vétérinaire, Université de Montréal, Saint-Hyacinthe, QC, Canada; ^2^Faculté de médecine et des sciences de la santé, Université de Sherbrooke, Sherbrooke, QC, Canada; ^3^Centre de recherche en santé publique de l'Université de Montréal et du CIUSSS du Centre-Sud-de-l'île-de-Montréal (CReSP), Montréal, QC, Canada; ^4^Public Health Risk Sciences Division, National Microbiology Laboratory, Public Health Agency of Canada, St. Hyacinthe, QC, Canada

**Keywords:** sentinel surveillance system, vector-borne disease (VBD), Lyme disease, decision tool, surveillance planning

## Abstract

**Objectives:**

With vector-borne diseases emerging across the globe, precipitated by climate change and other anthropogenic changes, it is critical for public health authorities to have well-designed surveillance strategies in place. Sentinel surveillance has been proposed as a cost-effective approach to surveillance in this context. However, spatial design of sentinel surveillance system has important impacts on surveillance outcomes, and careful selection of sentinel unit locations is therefore an essential component of planning.

**Methods:**

A review of the available literature, based on the realist approach, was used to identify key decision issues for sentinel surveillance planning. Outcomes of the review were used to develop a decision tool, which was subsequently validated by experts in the field.

**Results:**

The resulting decision tool provides a list of criteria which can be used to select sentinel unit locations. We illustrate its application using the case example of designing a national sentinel surveillance system for Lyme disease in Canada.

**Conclusions:**

The decision tool provides researchers and public health authorities with a systematic, evidence-based approach for planning the spatial design of sentinel surveillance systems, taking into account the aims of the surveillance system and disease and/or context-specific considerations.

## Background

The geographical distribution of vector-borne diseases (VBDs) is increasing all around the world; some VBD are re-emerging in areas where they had disappeared for some time (e.g., malaria in Asia) whilst others are appearing in new locations (e.g., West Nile Virus in North America) (1, 2). Factors including climate change and globalization have expedited the process of disease emergence, as they have created favorable conditions for these diseases to evolve (2). As these facilitating factors are impossible to control in a timely fashion to stop and reverse geographical expansion of VBDs, public health authorities must adapt their practices and act further down the line of disease emergence– in preventing the transmission of pathogens from vectors to human populations.

To implement timely and efficient against VBDs, public health authorities require surveillance systems which provide a defined spatio-temporal portrait of the disease and vectors on their territory, over a time period of sufficient length to assess trends and intervention outcomes. Concurrently, for surveillance system to stay sustainable, the surface area and granularity of the surveillance performed are limited by finite resources, requiring that specific areas be prioritized when the whole of the territory cannot be fully surveyed. This issue is further emphasized when disease prevalence increases. This phenomenon imposes additional stress on surveillance systems and resources, which may further restrict surveillance coverage of the study area e.g., passive tick surveillance in Canada, which was gradually reduced in endemic regions (3).

Sentinel surveillance offers the opportunity to target specific locations to inform about risk across larger study areas, thus reducing resources required by limiting sampling units and effort (3). Sentinels are a finite subunit of a population which are measured repeatedly through time. However, as the sample size is restricted, the sentinel units and their locations must be carefully chosen during the planning phase to effectively answer to surveillance objectives and avoid suboptimal use of resources or even inaccurate results. For instance, some locations may be better suited to following disease and pathogen trends, while others may be more effective at capturing early warnings of disease emergence. Furthermore, if vector surveillance is carried out in ecologically unsuitable environments, absence of vectors may falsely indicate low risk of VBDs across the surveillance zone.

In the context of VBDs, sentinel surveillance has been both successful and unsuccessful in monitoring disease risk for human populations. In some cases, the use of sentinel animals (e.g., chicken, horse, crow) has allowed for early detection of West Nile virus; however, this has not always been replicated and sentinels occasionally fail to emit a signal prior to the diagnosis of the first human cases (4–8). Dogs can also serve as effective sentinels to track the risk of Lyme disease (LD) in endemic regions, but research has shown that in non-endemic regions, canine seroprevalence is not a representative measure of the risk to humans in the context of emergence (9–11). These examples highlight the complexity of decision-making in sentinel surveillance for VBDs and the fact that although surveillance may work in a particular setting, the application of the same protocol may not be effective in another context.

One of the first decisions to be taken by public health authorities in establishing a sentinel surveillance system is to determine which type of sentinel unit will be used. We will define a sentinel unit as the statistical unit of the surveillance system associated with a known geographical location. As such, sentinel units can be diverse and include individual animals, animal herds, medical/veterinary clinics, physicians, laboratories, zoos, etc. To support researchers in choosing the right sentinel species, a framework has been previously generated (12). Once the type of sentinel unit has been chosen, it must be distributed spatially across the study zone. The importance of geographical location of the sentinel units for the effectiveness of the sentinel system has been highlighted in a previous framework (13). For sentinel surveillance of influenza, the WHO has established guidelines for selection of sentinel sites (14). However, such guidelines (or similar decision tools) are missing for sentinel surveillance of VBDs.

In Canada the emergence of Lyme disease is a public health priority (15, 16), and a national sentinel surveillance network for active acarological surveillance is being implemented. However, to ensure effective surveillance across a large study zone, a decision tool to support the selection of geographical locations of sentinel sites (from here on, this concept will be referred to as sentinel unit locations) should be utilized to ensure a systematic approach to surveillance system design; such an approach would ensure reproducibility of the surveillance design and homogeneity in the decision-making steps, encouraging comparability of results. In response to this problem, our research team previously conducted a scoping review to extract selection criteria used in choosing sentinel unit locations across different epidemiological contexts. As epidemiological context and surveillance objectives may influence spatial design of a sentinel surveillance system, we identified the need for a systematic approach to ensure key decision issues are addressed during the planning phases of the surveillance system.

The first aim of this study was to develop a decision aid tool to support the selection of sentinel unit locations, by identifying relevant criteria to consider for geographical distribution of sentinel units within the study zone. The second aim was to demonstrate the functionality of the decision tool by applying it to the design of a national sentinel surveillance system for emerging LD risk in Canada as a case study. Our research will support public health authorities in transparent decision-making for planning of sentinel surveillance of VBDs, allowing the integration of spatially explicit information in surveillance design (17).

## Methods

### Development of the decision tool

To identify the decisional requirements to include in the spatial design of a sentinel surveillance system, based on the context of the surveillance initiative, we carried out a review based on a realist approach. Realist reviews have been used in the past to develop the conceptual basis and operations requirements for surveillance frameworks in vector-borne diseases, as these are designed to gain an understanding of how complex programs work in different settings (18, 19). We adapted this approach to meet our review needs, to allow us to evaluate how different criteria for choosing sentinel site locations are used in different contexts.

A recent scoping review provided the scope and the exploratory background search for this current review (20). The database of articles built up during the scoping review was used for the purposive sampling steps, as the search strategy corresponded to the need of our review (20). The primary studies were appraised to extract key decision points related to sentinel surveillance planning. These findings were synthesized and integrated as foundational aspects of the decision tool. Full details of the realist-type approach are provided in the Supplementary material 1.

Planning a surveillance system is a complex problem which requires several important decisions. Firstly, the type of sentinel unit should be decided upon e.g., site where vector surveillance will take place or where animal herd will be positioned, or a medical/veterinary clinic. Our decision tool will provide insight into how to distribute sentinel units across the study zone. However determining the number of sentinel units which will form the sentinel surveillance system are beyond the scope of the tool. Public health authorities and researchers may decide on this point based on resources available, surveillance objectives, and disease situation.

### Validation of the decision tool

To ensure the functional validity of our decision tool, 14 experts in public health surveillance of VBDs were contacted and asked to assess the tool for functionality, as done in previous methodological research (21). Experts were required to complete an individual web-based questionnaire, with the aim of assessing whether the proposed tool was relevant, complete, and self-explanatory. At the end of the questionnaire, text boxes were available for final comments and suggestions (Supplementary material 2). A total of six experts responded (43% response rate) and questionnaires were examined by the research team and the results were used to update and improve the decision tool. The final version includes findings from our literature analysis and modifications following the validation by experts.

### Application of the tool: A case study

Lyme disease cases in Canada have shown a significant expansion during the 21st century; since its addition to the notifiable disease registry, the number of reported cases went from 144 cases reported in 2009 to close to 3,000 cases in 2021 (22). In response to this increasing risk, the Canadian Lyme Disease Research Network (CLyDRN) was created. As part of its research objectives, the CLyDRN had the mandate to build a sentinel surveillance system to provide comparable LD risk measures across the country, based on active surveillance of ticks. A sentinel approach was advocated as it allows for a feasible surveillance strategy across a vast study zone. The selection of criteria to guide final geographical location of sites for this LD sentinel surveillance system at a national level was used as a case study to illustrate the application of the decision tool in supporting selection of sentinel unit locations.

## Results

### Identification of a decision path and key decisions issues

In the previous scoping review, criteria were classified into six categories: past information, risk, environment, human population characteristics, distribution of sites, and logistics (20). This classification was kept as a skeleton for the decision tool and was used to identify key decisions that should be considered during sentinel site selection, to account for fundamental aspects of the epidemiological situation. The decision issues identified for each criterion, which constituted a key element of the tool were identified from the review (Table 1).

**Table 1:** Selection criteria for choosing sentinel unit locations within a surveillance system for vector-borne diseases; each criterion has associated decision issues which public health authorities or academics must consider in light of their surveillance context.

**Criteria group**	**Criterion**	**Decision issues**	**Selected references**
**Past information** Previous knowledge, from former studies or surveillance programs, which support the selection of sentinel unit location	Sites used in previous studies or surveillance initiatives	•These sites can provide a longer temporal series •Data from these sites could provide valuable insight into the current situation of the disease within the sites	(23–33)
	Sites with previous interventions	•When testing public health interventions, there must have been documented interventions conducted at the sentinel sites; this can be a single or multiple types of interventions /intervention intensities	(34–42)
**Risk** The presence or absence of an indicator of risk or use of a measure of risk to determine priority areas for sentinel unit location	Risk measure from host animals	•For Early Warning System (EWS), risk measures from host animal data are commonly used to select sentinel sites •A combination of human case, vector and host animal data can be used to evaluate risk level if following disease or pathogen trends	(8, 43–48)
	Risk measure from vector data	•Vector data, such as abundance of vectors, is often used to provide a measure of risk to target sentinel regions •May be appropriate in the context of EWS •A combination of human case, vector and host animal data can be used to evaluate risk level if following disease or pathogen trends	(24, 44, 45, 48–51)
	Risk measure from human case data	•Human case data can be used to target zones of higher risk and identify priority regions which should be monitored by sentinels •For EWS using a risk measure from human case data doesn't provide a timely signal •A combination of human case, vector and host animal data can be used to evaluate risk level if following disease or pathogen trends	(23, 43, 45, 52–54)
	Variation in risk	•When the purpose of the surveillance system is to test an intervention method, having sites with a variety of risk levels can evaluate intervention efficacy across different epidemiological contexts	(55–62)
**Environment** Consideration of the ecological features of the study zone to determine priority areas for sentinel unit location	Ecology suitable for vectors	•Appropriate ecology for the establishment of vectors is a prerequisite for VBD circulation	(24, 49, 63–68)
	Consideration of geographical features	•Certain geographical considerations, such as altitude and latitude, can be determinants of presence of VBDs	(23, 29, 45, 47, 59, 66, 69–71)
	Variation in ecological features	•A variation of ecological features across sentinel unit locations may be required if the surveillance system involves risk factor profiling	(6, 38, 55, 72–78)
**Human population** Human population characteristics are used to determine priority areas for sentinel unit location	Consideration of population numbers or population density	•Surveillance systems will attempt to maximize their population coverage	(45, 53, 56, 79–86)
	Population demographics	•Population demographics can influence VBD pathogen cycles e.g., population structure •To target sentinel unit locations which are relevant to the surveillance objectives, considering population demographics may be of benefit e.g., targeting areas where high-risk groups reside	(56, 57, 83, 87, 88)
	Population movements	•In some disease contexts, population movements are important to consider as they support a better understanding of the epidemiological portrait •E.g., individuals emigrating from an area endemic for malaria may facilitate spread of the parasite across locations •E.g., mechanical movements of humans could bring vectors e.g., mosquitoes	(36, 81, 85, 87, 89–91)
	Presence of human activities	•Depending on disease context, consideration of human activities can be important in the surveillance context	
		•E.g., human activities in aquatic environments are required for the transmission of schistosomiasis •E.g., outdoor activities can increase exposure to vectors	(44, 92–94)
**Distribution of sites** Spatial considerations for distribution of sentinel units across the study area	Administrative boundaries	•To ensure equity of resource allocation, it may be desirable to consider administrative boundaries (municipal, regional, etc.)	(66, 73, 84, 95–103)
**Logistics** Feasibility of the sentinel surveillance system, including access or diffusion of results	Site accessibility, voluntary participation, communication facilities, health centers, etc.	•To ensure sustainability and feasibility of the surveillance system, logistic criteria should be considered	(34, 53, 68, 81, 104–119)

The starting point for the tool is to consider any previous studies (*past information*) or unit locations which have been used in the predetermined study area. We propose to include these sites as a starting basis if they have been used to answer objectives similar to those of the sentinel system being developed. This will contribute to a longer temporal sequence for surveillance and previously collected data could provide valuable insight into the current situation of the VBDs within the sites (23–33). Next, if the objective of the system will be to evaluate a public health intervention, it is important to know whether there have been previous interventions conducted within the sites and choose sites accordingly (34–42).

The next category of criteria to consider is *risk-level*, that is, the presence or absence of an indicator/measure of risk to determine priority areas for sentinel unit locations. Sentinel sites are often sampled using a risk-based approach targeting subgroups of a population where disease or the pathogen is more likely to be present (13). Many different types of sources of data can be used for evaluating risk to humans, e.g., using data from vectors, host animals, or human cases (23, 24, 43–45, 49, 52–54). Often, these can be integrated together to obtain an overall risk signal. Publicly available databases e.g., the Expanded Special Project for Elimination of Neglected tropical diseases (ESPEN) (120), can provide large scale risk data and can be used to understand the variation in risk across space. Early warning systems (EWS) constitute a special case for which human case data may not provide a signal in a timely manner and for which other data sources, such as host animal data should be prioritized; their use has been frequently reported in the literature and has resulted in sensitive surveillance systems (8, 43–48). Another valid alternative is vector data, including vector abundance or pathogen prevalence in vector populations. Data availability and accessibility may affect the selection of risk-based criteria.

Because *environment* plays such a crucial role in the transmission cycle of vector-borne pathogens, it constitutes an important category and criteria pertaining to it are involved in key decisions issues. Ecological suitability for presence of vectors and climatic conditions are predominant criteria (24, 49, 63–67). Larger variation in ecological features may allow for risk factor profiling (6, 38, 55, 72–78). For use of surveillance systems as EWS, we recommend that the selection criteria be orientated toward a risk-based measure, as opposed to environmental criteria, to improve specificity.

In public health surveillance, population-oriented approaches are advocated. To get the best representativeness, surveillance system will aim to maximize population coverage (45, 53, 56, 79–86). Other *human population* criteria which may be utilized by the researcher are dependent on the surveillance objectives and disease context (56, 57, 83, 87, 88). For instance, does the surveillance initiative target a particular population structure? Is population stability of key importance in the transmission cycle, as seen in lymphatic filariasis (36, 81, 85, 87, 89–91)? is the presence of certain human activities required for disease transmission, for instance human water reservoir contact for schistosomiasis (44, 92–94)?

In the *distribution* of site category the main criterion identified was equity of resources allocation for distribution of sentinel units across the study area. For instance, although risk may be concentrated in a particular area, it may be necessary to characterize and follow the risk in different areas. Administrative boundaries (e.g., municipal, county, or regional) can be used to ensure equity and presence of a sentinel unit in different or priority administrative sectors (66, 73, 84, 95–103).

*Logistics* criteria were incorporated as a decision step within the decision path to enhance the feasibility and sustainability of the system. This last group of criteria can also be used as a discriminatory feature to select between multiple potential sites which are equal in terms of the previous selection criteria. This group of criteria mostly deals with any logistical constraints related to the sentinel unit location including the need of voluntary participation, presence of specialist centers, stakeholder opinions, or adequate communication facilities (34, 53, 68, 81, 104–119).

### A decision tool for sentinel surveillance of vector-borne diseases

**Figure 1:**
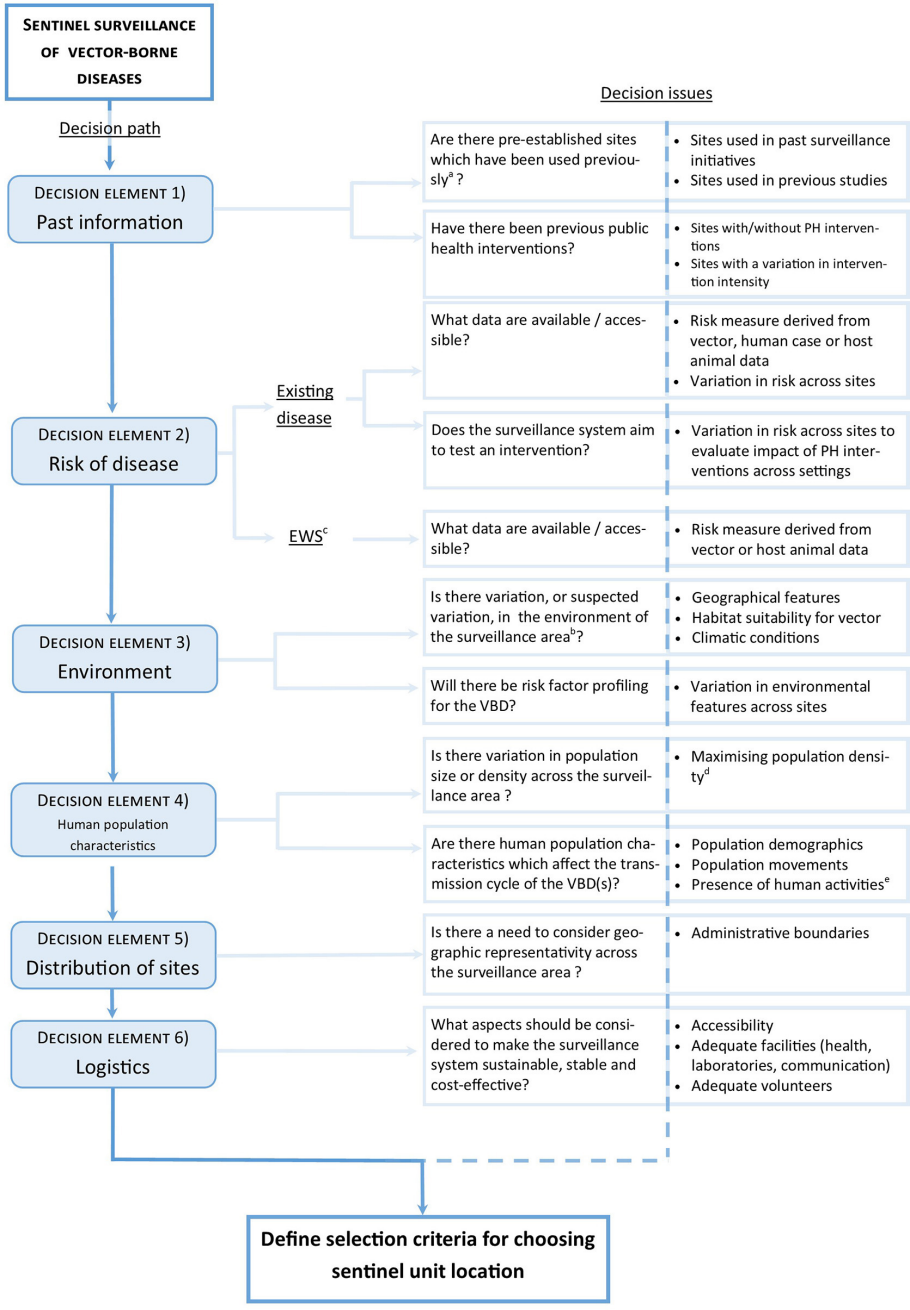
Decision tool for determining key criteria in developing a protocol for the selection of sentinel unit locations for vector-borne diseases. ^a^Site should have been used for a similar objective. ^b^The variation in the environment is judged significant by the investigators. ^c^Early warning system. ^d^It is also relevant to consider potential important population influx e.g., from tourism, occupational reasons. ^e^Human activities which influence exposure to vectors/vector-borne diseases.

Broad criteria categories were organized in a decision path to form a logical sequence of checkpoints and act as the tool for criteria selection. The user can follow each step of the path, however it may be used in an iterative manner. Within each step, key considerations identified through the review are presented as decision issues; these strategic questions can be answered by users during the planning process. Finally, the decision tool was assessed by experts and adjusted accordingly to ensure its validity (Figure 1). The functionality of the decision tool is demonstrated using a case study (section Sentinel surveillance for Lyme disease in Canada: A case study).

### Sentinel surveillance for Lyme disease in Canada: A case study

The objective of the Canadian Lyme Sentinel Network (CaLSeN) is to follow spatiotemporal LD risk trends in Canada. Following the decision to build a sentinel surveillance network, the Surveillance Working Group began by deciding upon the basic network structure. The sentinel unit within the network will consist of a sentinel region, where active surveillance efforts (drag sampling for ticks) will be concentrated. Sentinel regions will consist of a 50 km radius-wide area in close proximity to a population center and will contain 5–10 individual sampling sites. LD risk is very different across provinces and to provide a comparative portrait of LD, at least one sentinel region will be selected in each province. This will ensure that all provinces are represented, meeting CLyDRN's mission statement. The number of sentinel regions will depend on the size of the province, which varies greatly, and on each province's capacity to carry out fieldwork (human resources). As part of the initial planning phases for the surveillance network, we should consider how the spatial design of the network will be constructed.

**Figure 2:**
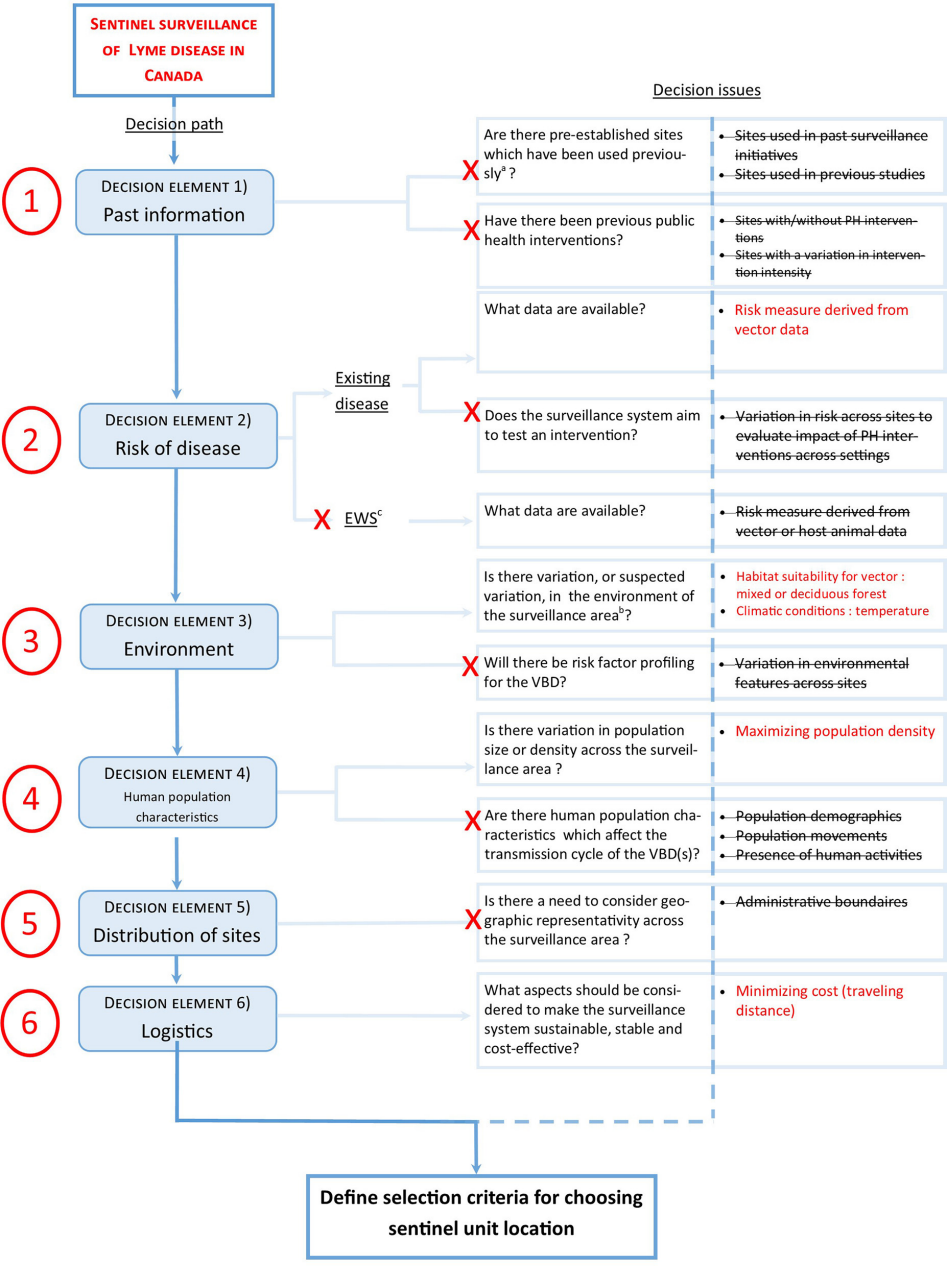
Demonstration of the functionality of the decision tool for determining key criteria for selecting spatial design for a national sentinel surveillance network for Lyme disease in Canada (case study). ^a^Site should have been used for a similar objective. ^b^The variation in the environment is judged significant by the investigators. ^c^Early warning system.

The decision tool was used to determine how sentinel regions will be distributed across Canada. The decision path was used (Figure 2). At the first checkpoint (*past information*), we considered if previous sites have been used in similar surveillance initiatives. Although there has been past active surveillance done in most of the Canadian provinces, there are no sentinel regions established for more intensive active surveillance initiatives. There are no planned public health interventions as part of the surveillance system. Thus, at this checkpoint, no criteria were retained. For the second checkpoint (*risk*), we are monitoring an existing disease, and the focus is not to act as an EWS but to provide a representative epidemiological portrait across Canada. Human case data are difficult to obtain, due to their sensitive nature, and we have resorted to passive tick submissions, available across the territory. Passive tick submissions have been determined to be a good signal for LD risk in human population in past studies (121). This was the only risk criterion retained, as currently no interventions are planned within the surveillance system. For the third checkpoint (*environment*), as we see important variations in environment-type within provinces, this criterion should be incorporated in the decision-making process. Furthermore, as climate change is an important factor for tick range expansion and tick population establishment, climate, in the form of temperature, was also retained. Risk factor profiling is not a primary aim of the surveillance system, hence variation in ecology was not kept as a criterion. For checkpoint 4 (*human population characteristics*), we wished to maximize the human population covered by the surveillance system i.e., we aimed to select sentinel regions with higher population density such as urban centers. We decided not to consider population characteristics, as access to demographic data at the municipal level across the whole of Canada posed challenges. Nonetheless, this could also be retained e.g., to consider populations with higher risk of exposure to black-legged ticks such as forest workers, indigenous communities, etc. (122, 123). For checkpoint 5 (*distribution*), the design of the sentinel system already determines how resources are allocated: we aim for at least one sentinel region per province. However, within each province, there is no need to consider administrative boundaries. Lastly, for checkpoint 6 (*logistics*), the main determinants of sustainability of the network are related to cost. Communication and laboratory facilities will not be impacted by the location of the sentinel region. Sampling material costs are not impacted by choice of sentinel region, but important variation travel costs and human resources will be associated with travel distance between CLyDRN collaborating centers and the sentinel region.

Using the decision tool, a total of five criteria were retained (Table 2). These criteria can subsequently be used within a multi-criteria decision analysis (MCDA). The MCDA encourages the participation of multiple stakeholders and provides a transparent decision-making approach. Such an analysis is the object of ongoing work in the context of this case study.

**Table 2:** Criteria to consider for planning the spatial design of a sentinel surveillance system for Lyme disease in Canada, retained after use of the decision tool.

**No**.	**Selection criterion**
1.	Measure of risk of Lyme disease as represented by passive acarological surveillance data
2.	Ecological suitability within the sentinel region for the presence of *Ixodes* spp., in the form of presence of mixed or deciduous forests
3.	Climatic suitability within the sentinel region for the presence of *Ixodes* spp., in the form of accumulated degree days
4.	Population density covered by the sentinel regions
5.	Traveling distance between the sentinel region and CLyDRN collaborating centers

## Discussion

Our study has permitted the development of a new decision tool to guide spatial design of sentinel surveillance systems. As sentinels are a limited sample of a population, careful selection of sentinel unit location is essential for the system to be effective. Although such decision tools were available for other types of infectious diseases, it was not the case for VBDs (124). As VBDs require complex interactions between pathogens, vectors and animal hosts, risk distribution becomes heterogenous in space (125). Careful selection of sentinel location becomes even more crucial to ensure data from sentinel sites is representative of the epidemiological portrait across the study area.

Conducting a review of the material obtained from previous work (20) allowed the identification of key decisions issues. We based our decision tool development approach on previous papers dealing with VBDs (13, 19). Being based on a broad literature search, a strength of our review was the inclusivity of research papers, providing a thoughout insight into key decision issues to consider for elaborating the spatial design of VBD sentinel surveillance systems. Despite this inclusivity, it is important to note that many papers in the literature do not explicit the decisional process behind selection of sentinel unit location; thus, these papers would have been excluded in the original scoping review database (20). Furthermore, some VBDs e.g., malaria, West Nile virus, are overrepresented (20). However, validation by experts working on different VBDs helped strengthen the decision path.

Vector-borne diseases represent a vast and heterogenous group of infectious diseases: their transmission cycles are complex and vary considerably from one disease to another. By keeping the scope of the decision tool wide, it is amenable to various VBDs but it could mean that some of the criteria suggested by the tool may not be relevant the specific disease or pathogen under surveillance. For instance, some VBDs do not rely on animal reservoirs e.g., malaria (126), whilst that others, such as West Nile Virus or LD, depend greatly on animal reservoirs to persist in the environment (127). Some criteria provided by the decision tool may be too broad for application and should be refined appropriately e.g., climatic conditions, habitat suitability. We acknowledge that to maximize the utility of the tool, users must have expertise in the field of the VBD under surveillance and also, knowledge about surveillance systems. Nonetheless, we believe the decision issues can be regarded as transversal: public health authorities or academics should follow the decision path regardless of the VBD(s) they are planning on surveying; this will ensure that key decision issues are not overlooked. The user must keep an open mind and flexible approach and use the tool as an aid as opposed to a strict procedural algorithm. The tool may be used in an iterative manner.

The inclusion of a vast scope of the literature has allowed the development of a decision tool that is not only adaptable to VBDs but also different contexts and surveillance objectives. However, the assessment of how the stage of the disease's emergence process may impact decision issues was not a focus of our realist-type review. The surveillance strategy may indeed diverge depending on whether a disease is absent, emerging or endemic. Although this could have an impact on sentinel unit locations, we would recommend first to evaluate the relevance of using a sentinel surveillance approach. Clow et al. (19) have developed a framework for adapting surveillance approaches across different stages of the emergence process. Such frameworks are complementary to our work and should be used conjointly during the planning phases of the surveillance systems.

In planning a public health surveillance system, the surveillance objectives should be decided on initially as these will have an impact on the system structure (128, 129). Using sentinel surveillance as an EWS can be a difficult endeavor; due to restricted sampling, sentinel surveillance has more often been used for monitoring temporal changes in frequently occurring diseases/pathogens or to detect disease outbreaks (129). Indeed, from our review, a very small proportion of studies had the aim of acting as an EWS (20); therefore, we recommend that the tool be used with caution if the aim of the sentinel surveillance system is to act as an EWS. In this case, we advise that the decision tool could be used alongside literature dealing with sentinel surveillance as EWS, specific to the VBD under investigation (13, 110, 130).

The functionality of the decision tool was demonstrated using our case example of building a new sentinel surveillance system for LD in Canada. Using the decision tool, we believe we were able to extract all relevant decision issues related to our case study. A total of five different criteria were retained from the decision tool (Table 2). Further use of the decision tool will contribute to validating its functionality, especially in differing contexts e.g., in developing countries, where access to data and research realities may be very different to the case study presented.

Although the tool does not integrate the relative importance of each criterion, additional processes can easily overcome this limitation. For example, MCDA have been used to address complex problems relating to vector-borne diseases, such as the development of intervention plans, where multiple and conflicting criteria are applied (17, 131). MCDA has also been used to map out risk areas for infectious diseases, such as avian influenza (132); we suggest that a similar approach could be utilized, in conjunction with criteria obtained from our decision tool, to identify sentinel locations. Indeed, MCDA is an inclusive, transparent, and systematic approach for incorporating different levels of information and could be used to integrate retained criteria from the decision tool in a practical manner.

Our decision tool has consolidated information from global VBD sentinel surveillance systems worldwide and channeled it into a methodical diagram which can aid in the selection process of sentinel unit locations in versatile circumstances. The selected criteria can be integrated in an MCDA model, allowing a participative approach with stakeholders concerned by the surveillance issue. In the future, the use of the decision tool in the establishment of sentinel surveillance systems for VBDs should be evaluated to demonstrate its operational strengths and limitations; new surveillance systems created with the support of this decision tool will require evaluation to provide additional insight into spatial design of sentinel surveillance for VBD for optimization of the decision tool.

## Data availability statement

The original contributions presented in the study are included in the article/Supplementary material, further inquiries can be directed to the corresponding author/s.

## Author contributions

CG: conceptualization, methodology, interpretation, writing original draft, review, and editing. CB and PL: supervision, conceptualization, methodology, interpretation, review, and editing. CA: conceptualization, methodology, interpretation, review, and editing. PB: interpretation, review, and editing. FM: supervision, review, and editing. All authors contributed to the article and approved the submitted version.

## Funding

This work was supported by the Canadian Lyme Disease Research Network (CLyDRN), supported by the Canadian Institutes of Health Research (CIHR), and the Faculty of Veterinary Medicine, Université de Montréal, Québec, Canada.

## Conflict of interest

The authors declare that the research was conducted in the absence of any commercial or financial relationships that could be construed as a potential conflict of interest.

## Publisher's note

All claims expressed in this article are solely those of the authors and do not necessarily represent those of their affiliated organizations, or those of the publisher, the editors and the reviewers. Any product that may be evaluated in this article, or claim that may be made by its manufacturer, is not guaranteed or endorsed by the publisher.
