# Supplementary material for: Criteria for selecting sentinel unit locations in a surveillance system for vector-borne disease: A decision tool
Source: Front Public Health. 2022 Nov 10;10:1003949. doi: 10.3389/fpubh.2022.1003949 (PMC9686450; doi:10.3389/fpubh.2022.1003949)
Supplement: Supplementary file 1 [file Data_Sheet_1.pdf]

Criteria for selecting sentinel unit locations in a surveillance system for vector-borne disease: A decision tool

Realist-type review protocol

Guillot Camille[*1,2,3], Bouchard Catherine[1,4], Aenishaenslin Cécile[1], Berthiaume Philippe[1,4], Milord François[2] & Leighton Patrick A[1,3]

[1] Groupe de recherche en épidémiologie des zoonoses et santé publique (GREZOSP), Faculté de médecine vétérinaire, Université de Montréal, Saint-Hyacinthe, Québec, Canada

[2] Faculté de médecine et des sciences de la santé, Université de Sherbrooke, Sherbrooke, QC

[3] Centre de recherche en santé publique de l'Université de Montréal et du CIUSSS du Centre-Sud-de-l'île-de-Montréal (CReSP)

[4] Public Health Risk Sciences Division, National Microbiology Laboratory, Public Health Agency of Canada, St. Hyacinthe, Québec, Canada

* Corresponding author: Camille.guillot@umontreal.ca

Based on:

Pawson R., Greenhalgh T., Harvey G., Walshe K. Realist review - a new method of systematic review designed for complex policy interventions. *J. Health Serv. Res. Policy*. 2005;10:21–34

Contents

Step 1: Classify scope 3

a. Identify the review question 3

b. Refine the purposes of the review 6

c. Articulate key theories to be explored 6

Step 2: Search for evidence 13

a. Exploratory background search 13

b. Progressive focusing to identify key programme theories 14

c. Purposive sampling 15

d. Final search for additional studies when review near completion 16

Step 3: Appraise primary studies and extract data 17

a. Use judgement to supplement formal critical appraisal checklists 17

b. Develop a ‘bespoke’ set of data extraction forms 17

c. Extract different data from different studies to populate evaluative framework with evidence 18

Step 4: Synthesize evidence and draw conclusion 18

Step 5: Disseminate, implement and evaluation 18

a. Draft and test out recommendations and conclusions 18

b. Work with practitioners and policy-makers to apply recommendations in particular contexts 19

c. Evaluate 19

References 20

Appendix 1 21

Our research team utilized a review approach based on the realist review (1) and modified the approach to suit our research objectives. All steps described below (Appendix 1) follow recommendations from Pawson *et al.* (1).

Step 1: Classify scope

a. Identify the review question

i. Nature and content of the intervention

The intervention under study for this realist review is the implementation of **sentinel surveillance systems for vector-borne diseases** (VBDs) of public health importance, as defined by the WHO (2). Some diseases determined to be of public health importance by the review team where included, despite not being on the WHO's list e.g., Bluetongue virus. Public health importance was determined by significant impact of human and/or animal health[1].

An initial scoping review (3) was used to list the various contexts where sentinel systems were used for surveillance of these VBDs.

ii. Circumstances or contexts for its use

A total of 36 various VBDs (or groups of VBDs e.g., tick-borne diseases) where surveyed across the articles retained in the scoping review (Table 1). The most frequently surveyed diseases included malaria, West Nile virus infection, lymphatic filariasis, and schistosomiasis.

Table 1. List of vector-borne diseases investigated within the articles included in the scoping review, including type of arthropod vector, type of pathogen and number of articles which studied each of these diseases

Disease	Vector	Pathogen	No. of articles
Malaria	Mosquitoes	Parasite	68
West Nile virus infection	Mosquitoes	Virus	32
Lymphatic filariasis	Mosquitoes	Parasite	22
Schistosomiasis	Snails	Parasite	19
Western equine encephalitis	Mosquitoes	Virus	15
Bluetongue	Midges	Virus	14
Murray Valley encephalitis	Mosquitoes	Virus	11
Onchocerciasis	Black flies	Parasite	11
Japanese encephalitis	Mosquitoes	Virus	10
Ross River virus	Mosquitoes	Virus	9
Arbovirus infection	Mosquitoes	Virus	7
Chikungunya	Mosquitoes	Virus	5
Zika	Mosquitoes	Virus	5

[1] Vector-borne diseases of plants were not captured with the search strategy

Barmah Forest virus infection	Mosquitoes	Virus	4
Yellow fever	Mosquitoes	Virus	4
Lyme disease	Ticks	Bacteria	4
Venezuelan equine encephalitis	Mosquitoes	Virus	3
Epizootic hemorrhagic disease	Midges	Virus	3
Arboviruses group A and B infection	Mosquitoes	Virus	2
Eastern equine encephalitis	Mosquitoes	Virus	2
Rift Valley fever	Mosquitoes	Virus	2
Utusu virus infection	Mosquitoes	Virus	2
Leishmaniasis	Phlebotomine sand flies	Parasite	2
Q Fever	Ticks	Bacteria	2
Saint Louis encephalitis	Ticks	Virus	2
Bovine trypanosomiasis	Tsetse flies	Parasite	2
Chaga's disease	Triatomine bugs	Parasite	1
Edge Hill virus infection	Mosquitoes	Virus	1
Jamestown Canyon virus infection	Mosquitoes	Virus	1
Bovine ephemeral fever	Midges	Virus	1
Schmallenberg virus infection	Midges	Virus	1
Bartonella infection	Ticks	Bacteria	1
Crimean-Congo fever	Ticks	Virus	1
Powassan virus infection	Ticks	Virus	1
Tick-borne diseases	Ticks	NA	1
African trypanosomiasis	Tsetse flies	Parasite	1

Other circumstances under which sentinel surveillance networks took place have been documented in the same scoping review, including:

1) Number of sentinel sites / number of locations where sentinels were placed (Table 2)
2) Number of years of operation (currently) of the sentinel surveillance system (Table 2)
3) Geographical location of the sentinel surveillance systems (Table 3)
4) Scale of operations of the sentinel surveillance systems (Table 4)

Table 2. Number of sentinel surveillance units in surveillance systems detailed in articles retained during a previous scoping review, according to the duration of the surveillance network operation.

Number	Duration of network operation (years)							Total
of sites	<1	1 to 2	3 to 5	6 to 10	11 to 20	>20	Unknown	
2	7	3	0	0	0	0	0	10
3-5	15	15	10	1	1	0	1	43
6-10	20	22	13	4	4	0	0	63
11-20	7	11	8	1	1	0	0	28
21-50	9	9	8	3	0	1	0	30
>50	3	3	2	3	1	8	0	20
Unknown	3	2	3	2	1	1	0	12
Total	64	65	43	14	8	10	1	206

Table 3. Geographical locations of sentinel surveillance systems detailed in articles retained during previous a scoping review and number of articles where the geographical location was elicited

Geographical location	Number of articles (%)
Africa	88 (42.7)
Asia	32 (15.5)
North America	27 (13.1)
Western Europe	18 (8.7)
Australia	15 (7.1)
Central or South America	13 (4.2)
Oceania	9 (3.9)
Eastern Europe	7 (3.4)

Table 4. Geographical scale of sentinel surveillance systems detailed in articles retained during a previous scoping review and number of articles where the geographical scale was elicited

Geographical scale of sentinel surveillance system	Number of articles (%)
Local	33, (16.0)
Regional	73 (35.4)
National	94 (45.6)
Multinational	6 (2.9)

The broad objectives of the sentinel surveillance systems in the articles retained in the previous scoping review included: following disease trends, testing intervention methods, profiling risk factors, and acting as an Early Warning System (EWS)[2,3] (Table 5).

Table 5. Objectives of sentinel surveillance systems detailed in articles retained during previous a scooping review, and number of articles which elicited each of the surveillance objectives

Objective of sentinel surveillance systems	Number of articles (%)
Following disease trends	128 (62.1)
Testing intervention methods	72 (35.0)
Profiling risk factors	32 (15.0)
Acting as an Early Warning System	15 (7.3)

iii. Policy intentions or objectives

[2] In the scoping review, some articles had the objectives of evaluating the sentinel surveillance system; as this is not an objective of the current realist review, which aims to investigate the establishment of new surveillance systems, this objective was not retained

[3] Some sentinel surveillance systems had more than one objective e.g., following disease trends and risk factor profiling

Our previous scoping review has allowed to describe the contexts in which sentinel surveillance systems have been developed for vector-borne diseases. These are diverse e.g., different geographical locations, different scales and different vectors/VBDs under surveillance.

The Canadian Lyme Disease Research Network (CLyDRN) has the mandate to construct a sentinel surveillance network for Lyme disease across Canada. The network will be constituted of sentinel nodes; these nodes will be defined as sentinel regions consisting of a circular area with a radius of 50km around a population center. Active surveillance in the form of drag flannel sampling will be conducted at sites within these regions.

A key consideration in planning the surveillance system was to decide where in space should the sentinel regions be located. There were no precise guidelines to answer this question, which lead to the following **review question**:

How to choose appropriate sentinel site locations for a sentinel surveillance system for vector-borne diseases according to the context?

b. Refine the purposes of the review

In this step, the purpose of the review is refined to capture an explanatory theme, based on a programme theory which has a clear impact on policy and can offer the potential for change. Pawson *et al.* (1) describe four different approaches:

1. **Theory integrity:** Purpose by theories of change evaluation, where complex programmes as viewed as sequence of stepping stones, each of which must be achieved successfully to reach the intended outcome
2. **Theory adjudication:** As many different interventions can be described in the literature, a realist review can uncover evidence to adjudicate between rival theories, or identify which permutation of mechanisms is most successful
3. **Comparison:** Here, it is assumed that programmes only work under certain circumstances and so, the review will uncover many studies of the 'same' intervention and can attempt to identify patterns of successful versus unsuccessful outcome.
4. **Reality testing:** This approach uses opposition between policy-makers and practitioners, grounds for political friction, to generate rival theories that may be put to empirical adjudication via a realist review.

For the current review, a **comparison approach** of the contents within the literature was used[4].

c. Articulate key theories to be explored

[4] As recommended by Pawson *et al.* (1), the final decision of which approach to use was finalized later during the review process; it is documented that realist review use iterative process and thus, pre-publication of realist review protocols are not recommended.

To set the stage for the realist review, the reviewers must familiarize themselves with current intervention theories found in the literature. From a long list of intervention theories, a short list will be drawn up and investigated in depth.

For our review question, which consists of:

How to choose appropriate sentinel sites locations for a sentinel surveillance system for vector-borne diseases?

published reviews have identified diverse criteria which have been used to select geographical locations of sentinel surveillance systems. In previous work, these criteria where extracted (Table 6).

Table 6. List of criteria used during planning the spatial design of sentinel surveillance systems and number of times different sentinel surveillance systems used each of the criteria

Criterion	Description	No. of articles
Risk (human)	There is documented risk of disease due to the presence of autochthonous human cases within the sentinel unit location (SUL)	55
Past surveillance	The SUL were chosen as they had been used in previously in surveillance programmes	34
Logistics	Logistical constraints (e.g., travelling distance, access) are considered for the SUL	31
Administrative boundaries	Selection of SUL according to administrative boundaries	29
Geographical features	The geography of the SUL has been taken into consideration during the selection	27
Variation in risk	There is a variation in degree of risk of the disease between the SUL	25
Variation in ecology	The SUL have been chosen due to variation in ecology between these units	23
Risk (vector)	There is documented risk of disease due to the presence of appropriate disease vector within the SUL	22
Previous studies	The SUL were chosen as they had been used in previously in scientific studies	21
Risk (unspecified)	There is documented risk of disease, however the nature of the risk is not elucidated within the SUL	20
Ecology (vector)	The ecology of the SUL is appropriate for the presence of the vector	18
Risk (host animals)	There is documented risk of disease due to the presence of appropriate host species within the SUL	17
Human population numbers	Selection of the SUL in order to maximize the human population reached within the units of the study zone	17
Random	Random distribution of SUL in the study zone	16
Voluntary	SUL are based on voluntary enrollment	14
Previous PH interventions	There are previous public health interventions carried out within the SUL	12
Population demographics	Population demographics are considered during the selection of SUL	10
Even distribution	Even distribution of SUL through the study zone	9
Proximity to risk	The SUL are in proximity to an area with document risk of disease	8
No previous PH interventions	There are no previous public health interventions carried out within the SUL	8
Variation in geography	The SUL have been chosen due to variation in geographical features between these units	7

Ecology (host animal)	The ecology of the SUL is appropriate for the presence of the host species	7
Ecology (unspecified)	The ecology of the SUL has been taken into consideration during the selection, however authors have not specified how	6
Population stability	The human populations within the SUL are stable (no immigration / emigration)	6
Risk (geography)	There is documented risk of disease due to geography (abiotic) within the SUL	5
Proximity to area of interest	The SUL is near an area of interest, such as a school	5
Variation in PH interventions	There is a variation in public health interventions carried out with the SUL	5
Livestock population	Selection of the SUL in order to maximize the volume of livestock within the units	4
Climate	Climate has been taken into consideration in the selection of the SUL	4
Suspected risk	There is suspected risk of disease within the SUL	3
Presence of human activity	There is presence of a specific type of human activity (e.g., fishing, hunting, wild mushroom picking) within the SUL	3
Areas of scientific interest	The SUL have been chosen as they represent areas of increased scientific interest	3
Stakeholders	The SUL are selected according to stakeholder preferences, suggestions, or recommendations	3
No risk	There is no document risk of disease within the SUL	2
Risk (interface)	There is documented risk of disease due to the presence of vector-human interface within the SUL	2
Ecology (disease)	The ecology of the SUL is appropriate for the presence of the VBD	2
Population instability	The human population within the SUL are unstable (immigration / emigration)	2
Minimal distance	Separation of SUL by a minimal distance	2
Specialist centers	There are specialists or a specialist centre within the SUL	2
Threshold of consultations	The SUL are selected in order to ensure that a minimal threshold of patient consultations is achieved	2
Variation in farming practices	The SUL have been chosen due to variation in farming practices between these units	1
Variation in housing type	The SUL have been chosen due to variation in housing type between these units	1
Health clinic demographics	Demographics of the health clinics are considered during the selection of SUL	1

Modeling	The SUL were chosen as there is modeling data to support their selection	1
Communications	There are adequate communication facilities within the SUL	1

Next, as part of the realist review process, previous criteria were grouped together based on the nature of each criterion (Table 7) and used to formulate general theories from which the review will explore in further depth. Afterwards, a theoretically-based evaluative framework was built as the backbone of the realist-type review (Figure 1).

Table 7. Criteria for selection geographical locations of sentinel units grouped by nature of the criterion

Group	Criterion	Description
Risk-level	Risk (human)	There is documented risk of disease due to the presence of autochthonous human cases within the sentinel unit location (SUL)
	Variation in risk	There is a variation in degree of risk of the disease between the SUL
	Risk (vector)	There is documented risk of disease due to the presence of appropriate disease vector within the SUL
	Risk (unspecified)	There is documented risk of disease, however the nature of the risk is not elucidated within the SUL
	Risk (host animals)	There is documented risk of disease due to the presence of appropriate host species within the SUL
	Proximity to risk	The SUL are in proximity to an area with document risk of disease
	Risk (geography)	There is documented risk of disease due to geography (abiotic) within the SUL
	Suspected risk	There is suspected risk of disease within the SUL
	No risk	There is no document risk of disease within the SUL
	Risk (interface)	There is documented risk of disease due to the presence of vector-human interface within the SUL
Environment	Geographical features	The geography of the SUL has been taken into consideration during the selection
	Ecology (vector)	The ecology of the SUL is appropriate for the presence of the vector
	Variation in ecology	The SUL have been chosen due to variation in ecology between these units
	Ecology (unspecified)	The ecology of the SUL has been taken into consideration during the selection, however authors have not specified how
	Variation in geography	The SUL have been chosen due to variation in geographical features between these units
	Ecology (host animal)	The ecology of the SUL is appropriate for the presence of the host species
	Proximity to area of interest	The SUL is near an area of interest, such as a school
	Livestock population	Selection of the SUL in order to maximise the volume of livestock within the units
	Climate	Climate has been taken into consideration in the selection of the SUL
	Ecology (disease)	The ecology of the SUL is appropriate for the presence of the VBD
	Variation in farming practices	The SUL have been chosen due to variation in farming practices between these units
	Variation in housing type	The SUL have been chosen due to variation in housing type between these units
Human population	Human population numbers	Selection of the SUL in order to maximise the human population reached within the units of the study zone
	Population demographics	Population demographics (e.g., population-level, health clinic) are considered during the selection of SUL
	Population stability	The human populations within the SUL are stable / unstable (immigration / emigration)
	Presence of human activity	There is presence of a specific type of human activity (e.g. fishing, hunting, wild mushroom picking) within the SUL
Distribution	Administrative boundaries	Selection of SUL according to administrative boundaries
	Random	Random distribution of SUL in the study zone

	Even distribution	Even distribution of SUL through the study zone
	Minimal distance	Separation of SUL by a minimal distance
Past information	Past surveillance	The SUL were chosen as they had been used in previously in surveillance programmes
	Previous studies	The SUL were chosen as they had been used in previously in scientific studies
	Previous PH interventions	There are previous public health interventions carried out within the SUL
	No previous PH interventions	There are no previous public health interventions carried out within the SUL
	Variation in PH interventions	There is a variation in public health interventions carried out with the SUL
	Areas of scientific interest	The SUL have been chosen as they represent areas of increased scientific interest
	Modelling	The SUL were chosen as there is modeling data to support their selection
Logistics	Logistics	All logistical consideration which will help the feasibility of the surveillance system, including:<br>- Logistical constraints (e.g., travelling distance, access) are considered for the SUL<br>- SUL are based on voluntary enrollment<br>- The SUL are selected according to stakeholder preferences, suggestions or recommendations<br>- There are specialists or a specialist centre within the SUL<br>- The SUL are selected in order to ensure that a minimal threshold of patient consultations is achieve<br>- There are adequate communication facilities within the SUL

Table 8. Theories developed, using preliminary literature search, to explain how spatial distribution of sentinel surveillance networks for sentinel surveillance systems have worked or not worked

Theory one	- Choosing sites where previous studies or previous surveillance initiatives have been done in the past can ensure that these sites are representative of the epidemiological portrait[5]
Theory two	- Evaluating risk level (using a known data e.g., vector densities, human case data) can assist in identifying sites of key scientific interest for surveillance of vector-borne diseases
Theory three	- Using environmental data can further assist in identifying sites of key scientific interest for surveillance of vector-borne diseases
Theory four	- As public health surveillance is population-orientated, considering human population densities is an important aspect for identifying sites of key scientific interest for surveillance of vector-borne diseases
Theory five	- Considering human population characteristics e.g., demographics, human activities, could be of particular importance for surveillance of specific vector-borne diseases
Theory six	- Using criteria to distribute sites across space could help to ensure that the entire study area is surveyed

[5] These sites should have served a similar surveillance objective

Theory seven	- Using logistical considerations could support feasibility and durability of the surveillance network

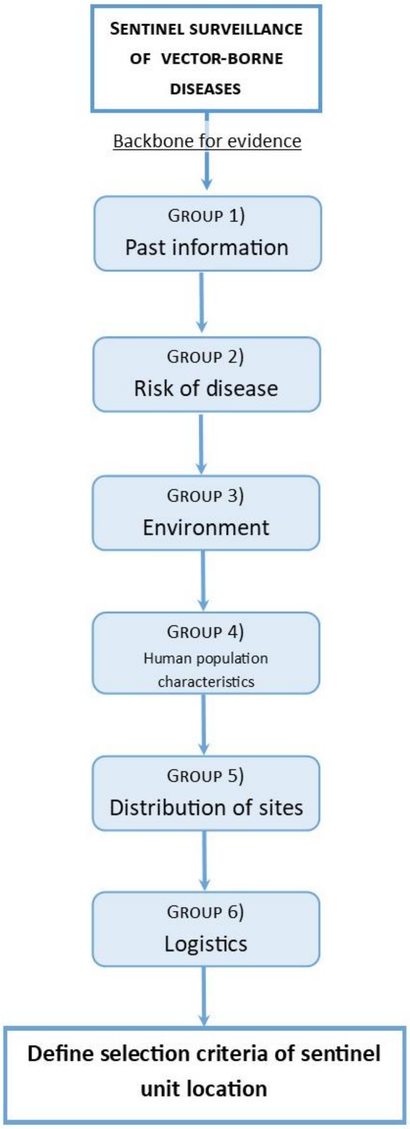

Figure 1. Evaluative framework for realist-type review; the order of the criteria groups was determined through discussion with review team and validated by experts in the field of vector-borne surveillance

Step 2: Search for evidence

a. Exploratory background search

The purpose of this step is to allow for getting a feel for the literature of the subject. This has been done during our previous scoping review (Figure 2). The search strategy used for the scoping review was developed to be inclusive: search terms related to (1) sentinel surveillance and (2) vector-borne diseases. Relevance screening was subsequently used on title and abstracts to keep only relevant articles.

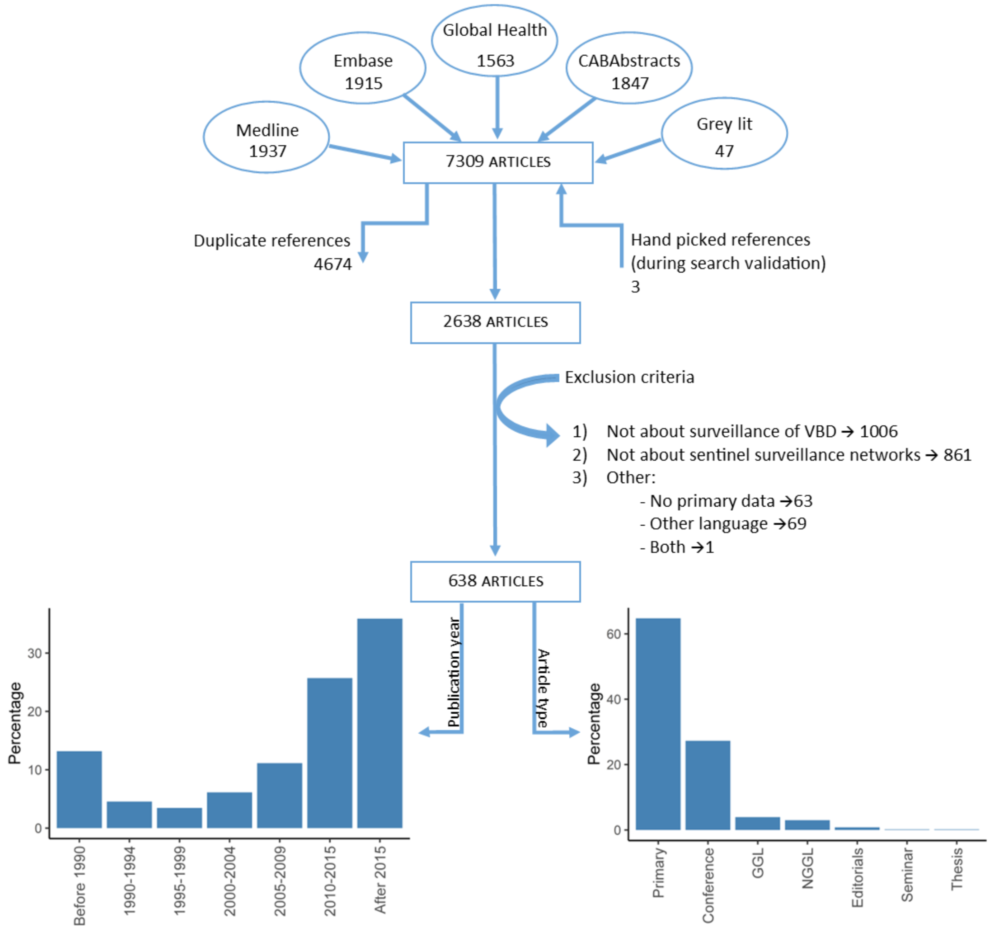

Figure 2. Search outcome from a scoping review aimed at investigating sentinel surveillance networks for vector-borne diseases, performed between September and November 2019. The search outcome is reported based on PRISMA guidelines. GGL, governmental gray literature; NGGL, non-governmental gray literature

b. Progressive focusing to identify key programme theories

Key program theories had been identified whilst speaking with key actors in the field (through CLyDRN surveillance group meetings) and through browsing of review papers including:

- Halliday JE, Meredith AL, Knobel DL, Shaw DJ, Bronsvoort BM, Cleaveland S. (2007). A framework for evaluating animals as sentinels for infectious disease surveillance. *J R Soc Interface*, 4(16):973-84.

- McCluskey, BJ. (2008). Use of Sentinel Herds in Monitoring and Surveillance Systems. 10.1002/9780470344866.ch8.
- Racloz V, Griot C, Stärk KD. (2006). Sentinel surveillance systems with special focus on vector-borne diseases. *Anim Health Res Rev* 7(1-2):71-9.

c. Purposive sampling

This has been described as the 'search proper' by Pawson *et al.* (1), where the reviewer moves on from browsing the literature (primary research), and a formal audit trail is provided. In the case of this review, a sensitive strategy was used to capture articles pertaining to sentinel surveillance for VBDs (Table 9). Database searched included CAB Abstract, Global Health and Embase and Medline. A total of 8 hours were spent looking at the gray literature.

Table 9. Search strategy for articles pertaining to sentinel surveillance and VBDs. The search strategy was modified for various databases

#	Searches
1	exp Disease Vectors/ or Tick-Borne Diseases/ or (vector* adj2 disease*).tw,kf,kw.
2	((arthropod* or insect* or mosquito* or aedes or anopheles or culex or tick? or triatomine bug* or sandflies or sandfly or sand flies or sand fly or blackfly or blackflies or flea? or triatomine bug* or tsetse fly or tsetse flies or aquatic snail*) adj2 (disease* or infect* or vector* or transmi* or fever* or borne or carrier* or carry or carries)).tw,kf,kw.
3	Chikungunya virus/ or Chikungunya Fever/ or chikungunya.tw,kf,kw.
4	exp Dengue/ or Dengue Virus/ or (dengue* or (fever adj2 (Aden or bouquet or breakbone or dandy or red or solar or sun))).tw,kf,kw.
5	Rift Valley Fever/ or (rift valley adj2 (fever* or virus*)).tw,kf,kw.
6	Yellow Fever/ or yellow fever.tw,kf,kw.
7	Zika Virus Infection/ or Zika Virus/ or (zika or (zikv adj2 (virus* or infect* or fever*))).tw,kf,kw.
8	exp Malaria/ or (malaria* or paludism* or swamp fever*).tw,kf,kw.
9	Encephalitis, Japanese/ or (encephalitis adj2 japanese).tw,kf,kw.
10	Elephantiasis, Filarial/ or (lymph* adj2 (filari* or elephantias*)).tw,kf,kw.
11	West Nile virus/ or West Nile Fever/ or ((west nile or "Egypt 101") adj2 (fever* or virus* or flavivirus* or disease*)).tw,kf,kw.
12	leishmaniasis/ or leishmaniavirus/ or (leishmani* adj2 (virus* or infect* or fever*)).tw,kw. or leishmanias*.tw,kf,kw.
13	Phlebotomus Fever/ or ((sandfly or pappataci or phlebotomus) adj2 (fever* or febris)).tw,kf,kw.

14	Hemorrhagic Fever Virus, Crimean-Congo/ or Hemorrhagic Fever, Crimean/ or ((crimean or congo) adj2 (virus* or infection* or fever* or h?emmorrhagic)).tw,kf,kw.
15	exp Borrelia Infections/ or (lyme* adj2 (disease* or borrelios*)).tw,kf. or (borrelia or borrelios* or (relaps* adj2 fever*) or neuroborrelios*).tw,kf,kw.
16	Q Fever/ or (coxiella burnet* infect* or coxiellos* or ((Q or query) adj2 fever*) or (rickettsial adj2 pneumoni*)).tw,kf,kw.
17	Encephalitis, Tick-Borne/ or Encephalitis Viruses, Tick-Borne/ or ((encephalit* or meningoencephalit*) adj2 (central european or tick or russian spring summer or forest spring or russian or vernal or tick or woodcutter* or louping ill or powassan)).tw,kf,kw.
18	Tularemia/ or (tular?emi* or francisella tularensis infect* or ohara disease* or yato bya).tw,kf,kw.
19	exp Trypanosomiasis/ or (trypanosomos?s or trypanosomias?s or trypanosoma infect* or african lethargy or sleeping sickness or nelavan or Chagas*).tw,kf,kw.
20	Plague/ or ((plague adj2 (bacterial or oriental)) or (yersinia adj2 pest*)).tw,kf,kw.
21	exp Rickettsia Infections/ or ((rickettsial* adj2 (disease* or infect*)) or rickettsios?s).tw,kf,kw.
22	exp Onchocerciasis/ or (onchocercias* or onchocercos?s or onchoceros?s or (onchocerca adj2 infect*) or river blindness* or robles disease* or onchodermatos?s or (onchocercal adj2 (skin* or derma* or cutaneous*))).tw,kf,kw.
23	exp Schistosomiasis/ or (schistosomias?s or schistomias?s or schistosomos?s or bilharzias?s or bilharzios?s or (schistosom* adj2 infect*)).tw,kf,kw.
24	Tick paralysis/ or (tick adj (paralys* or toxicos*)).tw,kf,kw.
25	Typhus, Epidemic Louse-Borne/ or Typhus, Endemic Flea-Borne/ or Typhus.tw,kf,kw.
26	or/1-25
27	Sentinel Surveillance/
28	(sentinel adj4 (surveillance or network* or system*)).tw,kw,kf.
29	Sentinel*
30	or/27-29
31	26 and 30

d. Final search for additional studies when review near completion

Using a snowball strategy, additional articles used during the construction of the decision tool included:

- Animal and Plant Health Inspection Service (APHIS) (2003). Bluetongue surveillance. The 2000 serological survey of slaughter cattle for antibody against bluetongue virus. Diagnostic Virology

Laboratory, National Veterinary Services Laboratories, Ames. Online: (aphis.usda.gov/vs/nahps/blue tongue/serological_survey.html. Accessed on 12 June 2022.

- European Council. (1992). Directive 92/119/EEC of 17 December 1992 introducing general Community measures for the control of certain animal diseases and specific measures relating to swine vesicular disease. *Off. J.,* L 062, 69-85.
- Hetzel MW, Pulford, J, Maraga S, Barnadas C, Reimer LJ, Tavul L, Jamea-Maiasa S, Tandrapah T, Maalsen A, Makita L, Siba PM, Mueller I. (2014). Evaluation of the Global Fund-supported National Malaria Control Program in Papua New Guinea, 2009-2014. *Papua and New Guinea medical journal*, 57(1-4), 7–29.
- Pearson JE, Gustafson GA, Shafer AL, Alstad AD. (1991). Distribution of bluetongue in the United States In Bluetongue, African horse sickness and related orbiviruses (T.E. Walton & B.I. Osburn, eds). Proc. Second International Symposium, Paris, CRC Press, Boca Raton, 128-139.
- Zhou G, Afrane YA, Vardo-Zalik AM, Atieli H, Zhong D, et al. (2011). Changing Patterns of Malaria Epidemiology between 2002 and 2010 in Western Kenya: The Fall and Rise of Malaria. *PLOS ONE*, 6(5): e20318.

Step 3: Appraise primary studies and extract data

a. Use judgement to supplement formal critical appraisal checklists

Realist reviews support the principle of evaluating data quality, as done in systematic review, however, utilize a different position. Whilst that systematic reviews evaluate data quality based on a strict hierarchy of evidence, this model limits greatly the information which can be obtained compared to a realist review. In comparison, during the realist review, multiple methods and approaches should be assessed to evaluate complex interventions.

Thus, the use of the investigator's judgement is the realist solution to quality control. The **relevance** and **rigour** of the retained articles are evaluated during this step (1).

Relevance: Relevance within a realist review is not about whether the study covered a particular topic, but whether it addressed the theory under test.

Rigour: Whether a particular inference drawn by the original researcher has sufficient weight to make a methodologically credible contribution to the test of a particular intervention theory

b. Develop a 'bespoke' set of data extraction forms

Conversely to systematic reviews, or even scoping reviews, realist review will assimilate information more by note-taking and annotation than by extracting data as such e.g., using data extraction forms. The aim of this

process is to identify theories within the retained articles, and whether the interventions (i.e., criteria using to select locations for sentinel units) have had successful outcomes (i.e., have reached surveillance objectives).

Thus, the database for note taking will contain the following headings:

Article name	Relevance	Rigor	Criteria used	Context	Theory

c. Extract different data from different studies to populate evaluative framework with evidence

The data from the different studies was collated into an Excel document.

Step 4: Synthesize evidence and draw conclusion

Step 4 can be summarized in four steps,

a. Synthesize data to achieve refinement of programme theory
b. Allow purpose of review (see Step 1b) to drive the synthesis process
c. Use 'contradictory' evidence to generate insights about the influence of context
d. Present conclusions as a series of contextualized decision points of the general format

These steps aim to determine what works for whom, how and under what circumstances, using the information obtained by step 3c). This have been done in an iterative manner to build a decision tool which will be presented as the final product.

Step 5: Disseminate, implement and evaluation

a. Draft and test out recommendations and conclusions

Once a 'final' decision tool, which incorporates recommendations and conclusions has been approved by the research team, it will be presented to experts and stakeholders for their opinion. The tool will be modified in consequence.

b. Work with practitioners and policy-makers to apply recommendations in particular contexts

This step is beyond the scope of the current article. However, its functionality will be illustrated though the use of a case example – for building a sentinel surveillance network in the south of Canada.

c. Evaluate

This step is beyond the scope of the current article. It will be a limit of the decision tool. To ensure its functionality and internal validity, sentinel surveillance programmes which develop using this tool will have to be evaluated.

References

1. Pawson ., Greenhalgh T, Harvey G, Walshe K. (2005). Realist review - a new method of systematic review designed for complex policy interventions. *J. Health Serv. Res. Policy*, 10:21–34

2. WHO. (2020). Vector-Borne Diseases. Available at https://www.who.int/news-room/fact-sheets/detail/vector-borne-diseases. .

3. Guillot C, Bouchard C, Berthiaume P, Mascarenhas M, Sauvé C, Villeneuve CA, Milord F, Leighton PA. (2021). A Portrait of Sentinel Surveillance Networks for Vector-Borne Diseases: A Scoping Review Supporting Sentinel Network Design. *Vector Borne Zoonotic Dis,* (11):827-38.

Appendix 1

Summary of key steps in realist review as described by Pawson *et al.* (1)

Step 1: Clarify scope<br>a. Identify the review question<br>• Nature and content of the intervention<br>• Circumstances of context for its use<br>• Policy intentions or objectives<br>b. Refine the purpose of the review<br>• Theory integrity – does the intervention work as predicted?<br>• Theory adjudication – which theories fit best?<br>• Comparison – how does the intervention work in different setting, for different groups?<br>• Reality testing – how does the intervention work in different setting, for different groups?<br>c. Articulate key theories to be explored<br>• Draw up a 'longlist' of relevant programme theories by exploratory searching (see Step 2)<br>• Group, categorize, or synthesize theories
Step 2: search for evidence<br>a. Exploratory background search to get a feel for the literature<br>b. Progressive focusing to identify key programme theories, refining inclusion criteria in the light of emerging data<br>c. Purposive sampling to test a defied subset of these theories, with additional 'snowball' sampling to explore new hypotheses as they emerge<br>d. Final search for additional studies when review near completion
Step 3: Appraise primary studies and extract data<br>a. Use judgement to supplement formal critical appraisal checklists, and consider 'fitness for purpose'<br>• Relevance – does the research address the theory under test?<br>• Rigour – does the research support the conclusions drawn from it by the researchers or the reviewers<br>b. Develop bespoke set of data extraction form and notation devices<br>c. Extract different data from different studies to population evaluative framework with evidence
Step 4: synthesize evidence and draw conclusions<br>a. Synthesize data to achieve refinement of programme theory – that is, to determine what works for whom, how and under what circumstances<br>b. Allow purpose of review (see Step 1b) to drive the synthesis process<br>c. Use 'contradictory' evidence to generate insights about the influence of context<br>d. Present conclusions as a series of contextualized decision points of the general format 'If A, then B' or 'in the case of C, D is unlikely to work'
Step 5: Disseminate, implement, and evaluate<br>a. Draft and test out recommendations and conclusions with key stakeholders, focusing especially on levers that can be pulled in here-and-now policy contexts<br>b. Work with practitioners and policy-makers to apply recommendations in particular contexts<br>c. Evaluate in terms of extent to which programmes are adjusted to take account of contextual influences revealed by the review: the 'same' programme might be expanded in one setting, modified in another and abandoned in another
