# Supplementary material for: Criteria for selecting sentinel unit locations in a surveillance system for vector-borne disease: A decision tool
Source: Front Public Health. 2022 Nov 10;10:1003949. doi: 10.3389/fpubh.2022.1003949 (PMC9686450; doi:10.3389/fpubh.2022.1003949)
Supplement: Supplementary file 2 [file Data_Sheet_2.pdf]

Criteria for selecting sentinel unit locations in a surveillance system for vector-borne disease: A decision tool

Guillot Camille[*1,2,3], Bouchard Catherine[1,4], Aenishaenslin Cécile[1], Berthiaume Philippe[1,4], Milord François[2] & Leighton Patrick A[1,3]

Supplementary material

Validation for decision tool

[1] Groupe de recherche en épidémiologie des zoonoses et santé publique (GREZOSP), Faculté de médecine vétérinaire, Université de Montréal, Québec, Canada

[2] Faculté de médecine et des sciences de la santé, Université de Sherbrooke, Sherbrooke, QC

[3] Centre de recherche en santé publique de l'Université de Montréal et du CIUSSS du Centre-Sud-de-l'île-de-Montréal (CReSP)

[4] Public Health Risk Sciences Division, National Microbiology Laboratory, Public Health Agency of Canada, St. Hyacinthe, Québec, Canada

* Corresponding author: Camille.guillot@umontreal.ca

Contents

1. Contact email for participation of experts 3
2. Introduction to the questionnaire 4
3. Original decision tool provided to experts 5
4. Case study provided to experts for demonstration of functionality of the decision tool 8
5. Questions within the questionnaire 10
6. Copy of the survey 11
7. Survey responses 15
8. Responses to comments and improvement of the tool 20
9. Improvement of the decision tool 25

1. Contact email for participation of experts

Below is the contact email which was sent to 11 experts to ask for their participation in validating the decision tool.

Dear all,

For those of you who don't know me, I am a PhD student in Dr Patrick Leighton's lab. My thesis looks at sentinel surveillance, in the context of vector-borne diseases, and more specifically for Lyme disease.

In the process of designing a surveillance system for vector-borne diseases, selecting the best sentinel sites can be challenging. In the context of my PhD, I have created a decisional tool which aims to help users to choose relevant criteria which can be used to objectively select such sites. The output of the decisional tool is a list of criteria, which can be used to determine where in space these sentinel sites should be located e.g., used as part of a multi-criteria decision analysis.

The tool was created with information gained from conducting a literature search, and data compiled to create a logical tool. It follows on from a previous article:

> Guillot C, Bouchard C, Berthiaume P, Mascarenhas M, Sauvé C, Villeneuve CA, Leighton P. A Portrait of Sentinel Surveillance Networks for Vector-Borne Diseases: A Scoping Review Supporting Sentinel Network Design. Vector Borne Zoonotic Dis. 2021 Aug 4. doi: 10.1089/vbz.2021.0008. Epub ahead of print. PMID: 34348055.

However, **this tool needs to be validated by experts** (academic and public health) to ensure its relevance, functionality, and completeness, before its publication.

I have created a questionnaire (about 30 mins) to allow for this assessment – your contribution would be greatly appreciated! In order to carry out the tool validation, please follow this link:

~~https://www.xxxxxxxxxxxxxxxxxxxxxx~~

Furthermore, the tool is available as a pdf attachment along with a case study which illustrates how to use the tool.

If you could complete the questionnaire by the **18th of October**, I would be very grateful.

Many thanks and don't hesitate to get in touch with any questions,

Camille

2. Introduction to the questionnaire

Below is the description of the task which was provided to experts for validation of the decision tool:

Hello,

Thank you for taking the time to complete this validation step for a new decisional tool.

In the process of designing a surveillance system for vector-borne diseases, selecting the best sentinel sites can be challenging. In the context of my PhD, I have created a decisional tool which aims to help users to choose relevant criteria which can be used to objectively select such sites. The output of the decisional tool is a list of criteria, which can be used to determine where in space these sentinel sites should be located e.g., used as part of a multi-criteria decision analysis.

The tool was created with information gained from conducting a literature search, and data compiled to create a logical tool. It follows on from a previous article:

Guillot C, Bouchard C, Berthiaume P, Mascarenhas M, Sauvé C, Villeneuve CA, Leighton P. A Portrait of Sentinel Surveillance Networks for Vector-Borne Diseases: A Scoping Review Supporting Sentinel Network Design. Vector Borne Zoonotic Dis. 2021 Aug 4. doi: 10.1089/vbz.2021.0008. Epub ahead of print. PMID: 34348055.

However, this tool needs to be validated by experts (academic and public health) to ensure its relevance, functionality, and completeness, before its publication.

I have created a short questionnaire (20-30 mins) to allow for its assessment. Please use the pdf sent to you by email in order to answer the questions.

Once again, many thanks and don't hesitate to get in touch with any questions,

Camille

3. Original decision tool provided to experts

The following decision tool (Figure 1) was provided in pdf form to experts. It was used to answer the questions within the online survey.

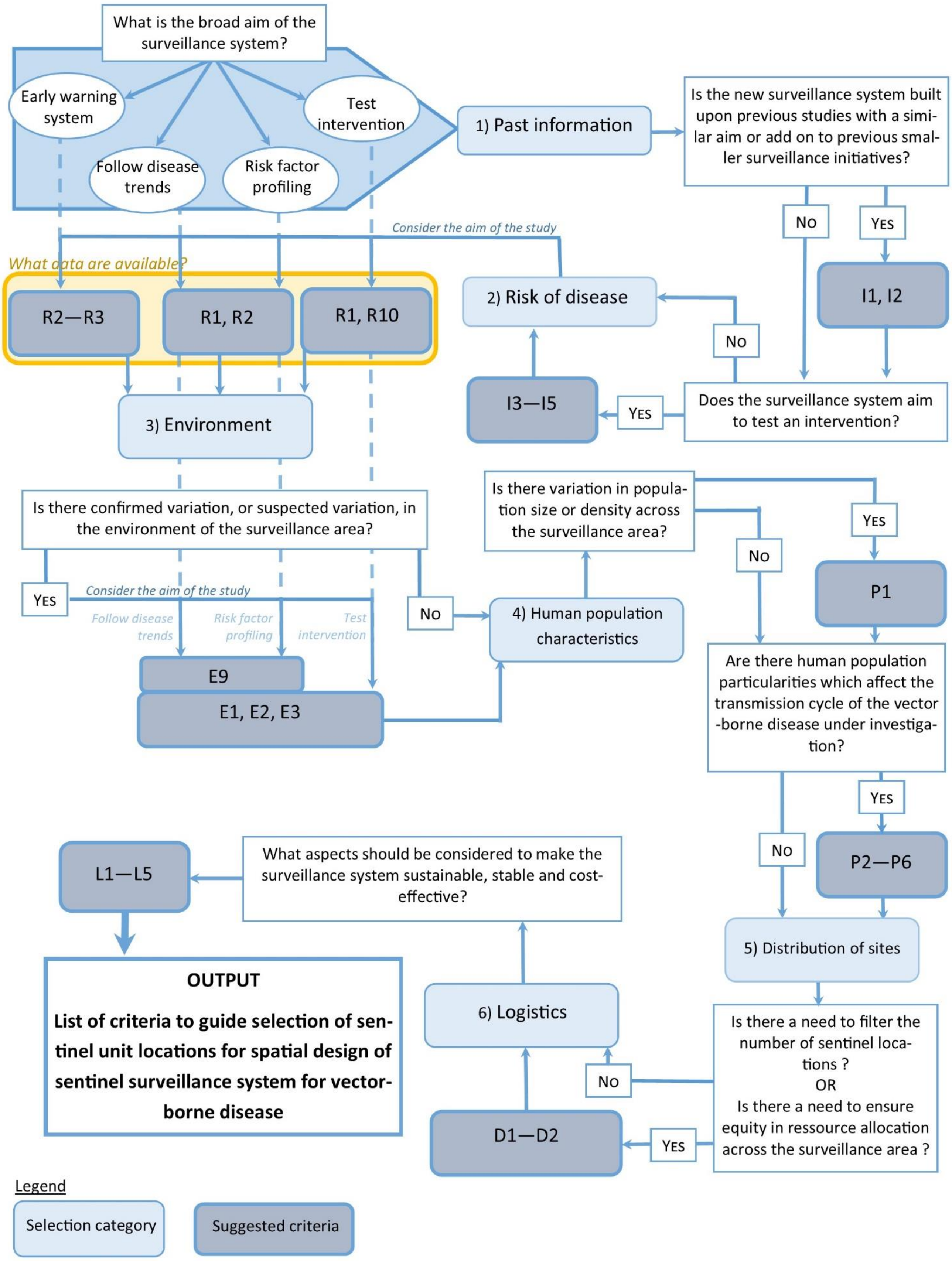
What is the broad aim of the surveillance system?
Early warning system
Follow disease trends
Risk factor profiling
Test intervention
1) Past information
Is the new surveillance system built upon previous studies with a similar aim or add on to previous smaller surveillance initiatives?
No
Yes
I1, I2
Consider the aim of the study
What data are available?
R2—R3
R1, R2
R1, R10
2) Risk of disease
No
I3—I5
Yes
Does the surveillance system aim to test an intervention?
3) Environment
Is there confirmed variation, or suspected variation, in the environment of the surveillance area?
Is there variation in population size or density across the surveillance area?
Yes
No
P1
Yes
Consider the aim of the study
Follow disease trends
Risk factor profiling
Test intervention
No
4) Human population characteristics
E9
E1, E2, E3
Are there human population particularities which affect the transmission cycle of the vector-borne disease under investigation?
Yes
No
P2—P6
L1—L5
What aspects should be considered to make the surveillance system sustainable, stable and cost-effective?
5) Distribution of sites
OUTPUT
List of criteria to guide selection of sentinel unit locations for spatial design of sentinel surveillance system for vector-borne disease
6) Logistics
Is there a need to filter the number of sentinel locations ?
OR
Is there a need to ensure equity in ressource allocation across the surveillance area ?
No
D1—D2
Yes
Legend
Selection category
Suggested criteria

D1 Consideration of administrative boundaries
D2 Random distribution of locations within the surveillance zone
E1 Consideration of the geography
E2 Presence of appropriate ecology for the presence of the vector
E9[1] Consideration of climate
I1 Selection of locations previously used in surveillance programs
I2 Selection of locations previously used in scientific studies
I3 Previous public health interventions carried out within the sentinel unit locations
I4 No previous public health interventions carried out within the sentinel unit locations
I5 Variation in public health interventions carried out across the sentinel unit locations
L1 Consideration of logistical constraints (e.g., traveling distance, access)
L2 Voluntary enrollment of sentinel unit locations
L3 Stakeholders' preferences, suggestions, or recommendations
L4 Presence of specialists or a specialist center within or near the sentinel unit location
L5 Presence of adequate communication facilities within or nearby the sentinel unit location
P1 Selection of the sentinel unit locations to maximize the population surveyed
P2 Consideration of population demographics (e.g., age, gender, socioeconomic status)
P3 Stability of human population (no immigration / emigration)
P5 Presence of a specific type of human activity (e.g., fishing, hunting, wild mushroom picking)
P6 Consideration of the patient demographics from participating the health clinics (e.g., age, gender, socioeconomic status)
R1 Presence of documented risk of disease, based on human case data
R2 Variation in degree of risk of the disease between the sentinel unit locations
R3 Presence of documented risk of disease, based on presence of appropriate disease vectors
R10 Presence of documented risk of disease, based on the evidence of vector-human contact
[1]criteria codes were kept from previous publication to keep these constant across our work

Figure 1. Decision tool provided to experts during tool validation step

4. Case study provided to experts for demonstration of functionality of the decision tool

Case study: Lyme disease at a national level

In North America, Lyme disease (LD) has been identified a priority VBD for public health due to its ongoing emergence, accelerated by climate change. In eastern Canada, the vector tick species, *Ixodes scapularis,* was first studied in the 70s at Long Point, Ontario and its range has shown ongoing expansion since this time. With Canada's vast territory, and the heterogeneous spread of tick populations in space, sentinel surveillance could allow a cost-effective, nationwide surveillance strategy to monitor the environmental risk of LD and track the risk through time. Hence, LD surveillance at a national level will be used as a case example to illustrate the application of the criteria selection tool in the determination of sentinel unit locations.

Such a surveillance system will be constructed by the Canadian Lyme Disease Research Network (CLyDRN) in the form of the Canadian Lyme Sentinel Network (CaLSeN). The aim of this system will be to follow trends in Lyme disease risk over time across Canada by evaluating the environmental risk of LD through active field surveillance of ticks. The sentinel unit will be a sentinel region, a geographical unit of 100km diameter around a population center. There will be minimally one sentinel region per province, and thus, the criteria selection tool will be used to determine which locations would be the most appropriate to act as sentinel regions at the provincial level.

Past information

Although there has been past active surveillance done in most Canadian provinces, they are no sentinel regions established for more intensive surveillance initiatives. Currently, there are no planned public health interventions as part of the surveillance system.

Risk

The primary aim of the sentinel surveillance system will be to follow trends in LD risk exposure in the environment. In the context of LD, although it was deemed a notifiable disease in 2009 in Canada, the human data case is owned by individual health boards and the geographic scale of the location of acquisition varies greatly between provinces. Due to these circumstances, data from passive surveillance is more easily accessible and remains the earliest signal of environmental risk of LD. Thus, for the system, the risk associated with the presence of the vectors will be used as a criterion for the selection of sentinel regional location.

Environment

Habitats vary greatly among and within provinces – from urban, barren land, wetlands, cropland, needle leaf forests, grassland, etc. (Canada Land Cover 2015). Many of these habitats will not be appropriate for the establishment of *Ixodes spp.* ticks, which require mixed or deciduous forests. Thus, this heterogenous land cover should be considered, and the presence of appropriate habitats for the establishment of vector populations should also be retained from the criteria selection tool. Furthermore, as the primary aim of the surveillance system is to follow disease trends, we can also select the criterion in which climatic features are considered, in this case temperature is the most relevant variable.

Population

Human population density varies greatly across provinces, with most of the population concentrated in urban centers. Thus, population density will be considered as a selection criterion. However, as LD remains a relatively rare disease in Canada, with an incidence of 2.7 cases / 100 000, population demographics or behaviors will not be considered.

Distribution

To ensure a minimum level of geographic representativeness, at least one sentinel region will be selected within each province, and up to three sentinel regions in the larger provinces that report higher incidences of LD, such as Ontario and Québec.

Logistics

As the system will cover a large geographical area, and that the system is intended to be maintained for many years to provide a longer temporal series, logistical aspects including costs of functioning are important to consider. Costs will vary with travel distance between research base and the sentinel region location. Materials, communication, and laboratory test results will stay constant regardless of the sentinel region location. Thus, to minimize costs and save time, travel distance between the sentinel region and the nearest research base should be considered during the selection of sentinel region location.

To summarize this case study, the criteria which researchers should use to select sentinel region location at the provincial level include:

1) There is documented risk of disease due to the presence of appropriate vector disease within the sentinel region (use of passive surveillance data)
2) Climate: considering temperature in the form of accumulated degree days.
3) The ecology of the sentinel region is appropriate for the presence of the vector (presence of mixed or deciduous forests)
4) Selection of the sentinel region to maximize the population reached within the units of the study zone
5) Logistical constraints (e.g., traveling distance) are considered when selecting sentinel regions

5. Questions within the questionnaire

The questionnaire comprised of a total of 14 questions.

1. Does category 1 (*Past information*) capture the required and relevant knowledge which should be considered initially, in order to build a surveillance system? (If not, please explain why)

2. Do you believe that use of surveillance aims / objectives to orientate which criteria should be selected from category 2 (*Risk of disease*) is relevant? (If not, please explain why)

3. Do you believe that the criteria suggested for category 2 (*Risk of disease*), according to surveillance aims, are relevant? (If not, please explain why)

4. For category 3 (*Environment*) do you think that the most relevant criteria have been included? (If not, please explain why)

5. Do you believe that the criteria suggested for category 3 (*Environment*), according to surveillance aims, are relevant? (If not, please explain why)

6. Do you believe that in building a public health surveillance system, population numbers / density should be considered? (If not, please explain)

7. For category 4 (*Population*), population particularities e.g., demographics, human activity, etc., are mentioned. The statement is broad in order to remain flexible and relevant for a variety of VBDs. Do you think this is appropriate and adequately formulated? (If not, please explain)

8. For category 5 (*Distribution of sites*), do you think there would be another reason, apart from filtering the number of sentinel locations or to ensure equity, do use this selection category? (If so, please explain why)

9. Category 6 (*Logistics*) has been included at the end of the decision tool in order to ensure that the surveillance system is sustainable. Do you agree with this step? (If not, please explain)

10. Do you think all relevant criteria, and relevant selection categories, are included within the decision tool? If not, which one(s) is(are) missing?

11. Is the decision tool clear and self-explanatory? Please write in the text box any suggestions to make the tool clearer or easier to use.

12. Is the flow of the decision tool logical? Please comment in the text box any suggestions on how to optimize the sequence of the tool.

13. Do you believe that the tool is flexible and adaptable to different vector-borne diseases in different geographical locations? (If not, please explain)

14. Please write any other comments or suggestions.

6. Copy of the survey

The online survey was carried out on LimeSurvey[1], as the principal investigator had access to a premier premium version through her Université de Sherbrooke affiliation. This version of LimeSurvey did not limit the number of respondents nor the number of questions which could be included. All questions were compulsory.

Validation of decisional tool for sentinel surveillance spatial design

Hello,

Thank you for taking the time to complete this validation step for a new decisional tool.

In the process of designing a surveillance system for vector-borne diseases, selecting the best sentinel sites can be challenging. In the context of my PhD, I have created a decisional tool which aims to help users to choose relevant criteria which can be used to objectively select such sites. The output of the decisional tool is a list of criteria, which can be used to determine where in space these sentinel sites should be located e.g. used as part of a multi-criteria decision analysis.

The tool was created with information gained from conducting a literature search, and data compiled to create a logical tool. It follows on from a previous article:

Guillot C, Bouchard C, Berthiaume P, Mascarenhas M, Sauvé C, Villeneuve CA, Leighton P. A Portrait of Sentinel Surveillance Networks for Vector-Borne Diseases: A Scoping Review Supporting Sentinel Network Design. Vector Borne Zoonotic Dis. 2021 Aug 4. doi: 10.1089/vbz.2021.0008. Epub ahead of print. PMID: 34348055.

However, this tool needs to be validated by experts (academic and public health) to ensure its relevance, functionality and completeness, before its publication.

I have created a short questionnaire (20-30 mins) to allow for its assessment. Please use the pdf sent to you by email in order to answer the questions.

Once again, many thanks and don't hesitate to get in touch with any questions,

Camille

There are 14 questions in this survey.

Next

* 1 Does category 1 (Past information) capture the required and relevant knowledge which should be considered initially, in order to build a surveillance system? (if not, please explain why)

Choose one of the following answers

- Yes
- No

Please enter your comment here:

* 2 Do you believe that use of surveillance aims / objectives to orientate which criteria should be selected from category 2 (Risk of disease) is relevant ? (if not, please explain why)

Choose one of the following answers

- Yes
- No

Please enter your comment here:

[1] https://www.med.usherbrooke.ca/limesurvey257/index2.php?r=admin

* 3 Do you believe that the criteria suggested for category 2 (Risk of disease), according to surveillance aims, are relevant ? (if not, please explain why)

Choose one of the following answers

- Yes
- No

Please enter your comment here:

* 4 For category 3 (Environment) do you think that the most relevant criteria have been included ? (if not, please explain why)

Choose one of the following answers

- Yes
- No

Please enter your comment here:

* 5 Do you believe that the criteria suggested for category 3 (Environment), according to surveillance aims, are relevant ? (if not, please explain why)

Choose one of the following answers

- Yes
- No

Please enter your comment here:

* 6 Do you believe that in building a public health surveillance system, population numbers / density should be considered ? (if not, please explain)

Choose one of the following answers

- Yes
- No

Please enter your comment here:

* 7 For category 4 (Population), population particularities e.g. demographics, human activity, etc., are mentioned. The statement is broad in order to remain flexible and relevant for a variety of VBDs. Do you think this is appropriate and adequately formulated ? (if not, please explain)

Choose one of the following answers

- Yes
- No

Please enter your comment here:

* 8 For category 5 (Distribution of sites), do you think there would be another reason, apart from filtering the number of sentinel locations or to ensure equity, do use this selection category ? (if so, please explain why)

Choose one of the following answers

- Yes
- No

Please enter your comment here:

* 9 Category 6 (Logistics) has been included at the end of the decisional tool in order to ensure that the surveillance system is sustainable. Do you agree with this step? (if not, please explain)

Choose one of the following answers

- Yes
- No

Please enter your comment here:

* 10 Do you think all relevant criteria, and relevant selection categories, are included within the decisional tool? If not, which one(s) is(are) missing?

Choose one of the following answers

- Yes
- No

Please enter your comment here:

Previous

Next

Overall assessment

* 11 Is the decisional tool clear and self-explanatory ? Please write in the text box any suggestions to make the tool clearer or easier to use.

Choose one of the following answers

- Yes
- No

Please enter your comment here:

* 12 Is the flow of the decisional tool logical ? Please comment in the text box any suggestions on how to optimise the sequence of the tool.

Choose one of the following answers

- Yes
- No

Please enter your comment here:

* 13 Do you believe that the tool is flexible and adaptable to different vector-borne diseases in different geographical locations ? (if not, please explain)

Choose one of the following answers

- Yes
- No

Please enter your comment here:

14 Please write any other comments or suggestions.

Previous

Submit

7. Survey responses

A total of 6 experts (43% response rate) responded to the questionnaire. Three experts (27%) contacted the principal investigator to explain that time constraints did not permit their participation.

Reviewer ID	Yes / No	Comments
1. Does category 1 (*Past information*) capture the required and relevant knowledge which should be considered initially, in order to build a surveillance system? (If not, please explain why)		
2	No	I'm not sure why this point is put first. For me, initial information would include: is the vector present here? In neighboring regions? Is there a risk of introduction? Are there cases? What is the impact? To see if it is useful or not to initiate surveillance and choose this type of surveillance. Additional, if a system already exists, it is not necessarily useful to do another one in.
3	Yes	
4	Yes	
5	Yes	
7	No	Even if selection of sites already known may seem like a good idea and could save time, their selection should not be systematic. Their selection should be dependent upon comparison with other possible options and 'pass the test' of this decisional tool, notably to validate that the selection criteria are in sync with the objectives. Instead of writing 'selection of locations in I1 and I2, I would write, 'consideration of locations'.
11	Yes	
2. Do you believe that use of surveillance aims / objectives to orientate which criteria should be selected from category 2 (*Risk of disease*) is relevant? (If not, please explain why)		
2	Yes	No comment
3	Yes	No comment
4	Yes	No comment
5	Yes	No comment
7	Yes	No comment
11	Yes	No comment
3. Do you believe that the criteria suggested for category 2 (*Risk of disease*), according to surveillance aims, are relevant? (If not, please explain why)		
2	Yes	Yes, however, should be more precise e.g., cases in domestic animals, wild animals or livestock, the risk of introduction of the vector / pathogen, specify the location of the risk, the abundance of the vector, host (animal or human)
3	Yes	
4	Yes	Relevant criteria included, seems thorough, I cannot identify other ones
5	Yes	
7	No	I'm not sure to understand R2 relative to the Early Warning System objective. For this same objective, we could also decide to detect the first appearance of a vector on the territory. In this case, R3 no longer applies. This depends on how early we want to be. The other criteria appear relevant with respect to the objectives
11	Yes	
4. For category 3 (*Environment*) do you think that the most relevant criteria have been included? (If not, please explain why)		
2	No	Other criteria can be including seasonality, preferential hosts, climatic and meteorological conditions for presence, activity and reproduction, conditions for the vector but also for the pathogen

3	Yes	
4	Yes	
5	Yes	
7	Yes	The description for criterion E3 is missing
11	No	Legend is missing E3 description.
5. Do you believe that the criteria suggested for category 3 (*Environment*), according to surveillance aims, are relevant? (If not, please explain why)		
2	Yes	The description for criterion E3 is missing
3	Yes	
4	Yes	
5	Yes	
7	Yes	I do not think it is clear if criteria E1, E2 and E3 apply to 'follow diseases trend and 'risk factor profiling'
11	No	E1 is a bit vague and should explain what geographical information is needed (for example topography, bioclimatic region, etc.).
6. Do you believe that in building a public health surveillance system, population numbers / density should be considered? (If not, please explain)		
2	Yes	It would depend on the surveillance objective: to detect the vector/pathogen/risk area or estimate the risk of transmission to humans. It also depends on the point of view e.g., a citizen who lives or walks somewhere versus public health who wants to know where there will be more cases
3	Yes	
4	Yes	
5	Yes	
7	Yes	The influx of people in a region for work or tourism should also be considered. A region could have a low population but be very touristic and represent a risk for the population
11	Yes	
7. For category 4 (*Population*), population particularities e.g., demographics, human activity, etc., are mentioned. The statement is broad in order to remain flexible and relevant for a variety of VBDs. Do you think this is appropriate and adequately formulated? (If not, please explain)		
2	Yes	
3	No	Appropriate: Yes<br>Formulation: I suggest 'under surveillance' instead of 'under investigation' for the tool to be more coherent
4	Yes	
5	Yes	
7	Yes	
11	No	P5 should be rephrased to be specifically about human activity susceptible to influence exposure, because many types of activities would not be relevant here, such as those indoors or without a strong component performed within natural settings. Also, I would use the widely recognized example of camping instead of wild mushroom picking.

	8. For category 5 (*Distribution of sites*), do you think there would be another reason, apart from filtering the number of sentinel locations or to ensure equity, do use this selection category? (If so, please explain why)	
2	Yes	On the whole study zone (if vector is already present) or a probable zone of emergence (borders / airport)
3	No	
4	No	
5	No	
7	Yes	Preference of local public health authorities (however this is included within the logistic criterion)
11	No	
	9. Category 6 (*Logistics*) has been included at the end of the decision tool in order to ensure that the surveillance system is sustainable. Do you agree with this step? (If not, please explain)	
		Cost, long-term management of the surveillance system, standardization of the protocol (data collection, laboratory analyses, data analyses, information key actors)
2	Yes	
3	Yes	
4	Yes	
5	Yes	
7	Yes	Evaluation of the impact level of each of these logistical aspects on the sustainability and functionality of the system. Ideally, do not make too many comprises relating to these criteria, to prevent deviating from the initial objective, unless this has an important impact on the quality of the system and the data. Overcome these logistical difficulties by finding alternative or finding measures to decrease these impacts, if possible.
11	Yes	
	10. Do you think all relevant criteria, and relevant selection categories, are included within the decision tool? If not, which one(s) is(are) missing?	
2	No	Type of data collected (vector collection? Serology? Human cases?)<br>Is there really a need for sentinel sites within the surveillance system? yes/no
3	Yes	
4	Yes	
5	Yes	
7	No	Incidence rate or abundance of ticks high enough to be able to meet the objectives (except early warning system). For example, if the number of ticks collected or the number of human cases is low, this could prevent trends from being identified due to small numbers and statistical uncertainties.
11	Yes	
	11. Is the decisional tool clear and self-explanatory? Please write in the text box any suggestions to make the tool clearer or easier to use.	
2	No	Objective of the decision tool should be well explained. Difficult to understand and follow without a text that accompanies the different steps and explains the logic. Not clear what we get at the end of the algorithm.
3	Yes	
4	Yes	
5	No	

7	No	Complex to understand, especially for someone who has never done surveillance site selection. The case study helps to understand: good idea. A presentation of the tool or an explanatory document could also have helped. At the end of the process, a question remains: which criterion(s) should be prioritized if we cannot find a sentinel unit corresponding to all these criteria? What do you suggest? Does the order of the criteria reflect the weight of the criteria? Could be translated in French (reviewer was francophone)
11	No	I just have some general formatting suggestions. It would be great if you could move the legend on the same page as the schematic, as it would make it a lot easier to go around it. Also, legend items do not have to be in alphabetical order and listing them in the order they are mentioned makes more sense. Also, avoid skipping numerical labels for easier reading. For example, we have R1 to R3 but skip to R10 after, so in this case I would switch the label R10 to R4, if this makes sense.
12. Is the flow of the decision tool logical? Please comment in the text box any suggestions on how to optimize the sequence of the tool.		
2	Yes	
3	Yes	
4	Yes	
5		
7	No	Work on the graph, especially the dashed arrows which make the trajectory confusing… maybe put in different colors instead?
11	Yes	
13. Do you believe that the tool is flexible and adaptable to different vector-borne diseases in different geographical locations? (If not, please explain)		
2	Yes	Probably because the categories are very broad, but not sure that it fits all vector-borne diseases, the algorithm seems more oriented towards Lyme
3	Yes	
4	Yes	
5		
7	Yes	
11	Yes	
14. Please write any other comments or suggestions.		
2	NA	
3	NA	Given that all the arrows (yes and no) arrive at the end of the algorithm, I wonder if an algorithm is really relevant in relation to a table that includes all the criteria to be taken into account to choose the sentinel sites
4	NA	
5	NA	
7	NA	
11	NA	Great job getting this done!

8. Responses to comments and improvement of the tool

Comments were analyzed (table 2) and regrouped into large points. These were addressed, and the decision tool was modified in consequence (Figure 2).

Comment from survey	Addressing the comment	Consequence on decision tool
Putting past information (from previous work) at the start of the decision tool may not be relevant	• 4/6 reviewers believe it to be relevant (potentially 5, as reviewer 7 does not question its place within the algorithm)<br>• From authors' previous experience, we believe that it is necessary to know what is already in place prior to building a new surveillance system<br>• However, important point it that the tool is not linear – this should be emphasized in the text<br>• Previous work (realist-type review) supports the use of these sites	• Nil
Selection of previous surveillance sites should not be systematic	• Old sites should be considered, however, also compared with new ones to ensure that sites are optimized<br>• Thus, this should be illustrated in the tool<br>• Also ensure that they meet surveillance objectives	• Create decision steps – these are not obligatory, however, identify key decision points that should be considered by decision makers (DMs)<br>• DMs must choose to accept or reject the criteria propositions
Criteria relating to risk should be more precise	• Several very relevant and specific criteria are provided e.g., cases in domestic / wild animals / livestock<br>• However, we believe these are too specific for the decision tool in itself; due to space and for usefulness, we are aiming for simplicity.<br>• We believe that the specificity suggested are present within broader criteria which are	• Nil in the decision tool itself; we aim to keep broader and more flexible so it can be applicable to a wide range of contexts<br>• However, this is a very important point which must be highlighted in the results section, how the tool was constructed, using concrete case

	suggested e.g., risk in animal hosts includes domestic / wild and livestock, and experts should refer to their knowledge of the VBD when using the tool<br>• In terms of location of the risk, this is inherent to the approach suggested by the paper – it does not seem relevant<br>• Hosts (animal or human) are separated into two categories (risk in human versus risk in hosts); however data availability constraints are also a very important limitation to the type of data which can be accessed and subsequently used	examples where specific risk criteria are developed
Presence of appropriate ecology for the presence of the vector relative to the Early Warning System (EWS) objective does not seem appropriate	• Very few examples of sentinel surveillance systems used as EWS for VBDs in the literature; due to few surveillance sites, it poses important logistical barriers to use this type of surveillance<br>• On second examination of realist-type review material, authors also agreed that this criterion is less relevant than others	• Risk criteria relative ecology was removed, and more general vector and host animal risk data were retained
Criteria relating to environment should be more precise	• Reviewer has suggested some important and precise criteria which could be considered<br>• However, we argue that these are included in larger criteria<br>• We believe these are too specific for the decision tool in itself; due to space and for usefulness, we are aiming for simplicity	• Nil in the decision tool itself; we aim to keep broader and more flexible so it can be applicable to a wide range of contexts<br>• However, this is a very important point which has to be highlighted in the results section, how the tool was constructed, using concrete case examples where specific risk criteria are developed
Application of criterion E1-E3 to other surveillance objectives	• Structure of the decision tool means it is not clear to what surveillance objectives these criteria should apply	• Change of the structure of the tool to make it easier to follow and know which criteria apply at which point
Description of E3 is missing	• The description is absent from the Legend	• Authors believe that this legend and the criteria ID is confusing, thus these have been removed from the figure;

		they are written out instead for ease of understanding
Criteria E1 'consideration of the geography' is a bit vague	• We aim for simplicity and flexibility of the tool<br>• But indeed, formulation of the criteria is confusing and is very broad<br>• More specific example should be given	• Change of formulation: geographical features<br>• Kept broad to allow flexibility; this requires users expertise relating to the disease under surveillance; it is an important limit which should be highlighted in the discussion<br>• Functionality will be highlighted within the results section (specific examples plus case example)
Consideration of population density depends on the surveillance objective	• Finding cases versus vectors is given as a case<br>• However, otherwise the rest of the reviewers agree that this is a relevant consideration<br>• Less likely to identify presence of vectors in a region where there is no human population – as there is no chance of exposure to the VBD and thus no risk<br>• Furthermore, the algorithm is for the use of public health authorities, using population-based versus individual-based approach	• Nil
Population flux relating to tourism and occupation can have an important impact on population density	• This can be very important for highly touristic areas, or for diseases where there is an occupational risk e.g., parks where park rangers are present	• Footnote added to the maximizing population criteria to account for population influx resulting from tourism / occupation
Formulation 'Are there human population particularities which affect the transmission cycle of the vector-borne disease under investigation?' is hard to understand	• Formation was deemed confusing<br>• Be rephrased to be specifically about human activity susceptible to influence exposure	• Formulation changed and footnote added to specify activities susceptible to influence exposure
Distribution of sites	• Reviewers overall thought it was relevant<br>• Some comments about criteria which are included elsewhere (e.g., relative to the distribution of the risk of disease, or relating to public health authority preference)	• Changed according to iterative inspection of realist-type review, as not many comments from reviewers

	• This decision criteria group was examined retrospectively, as it may not necessarily to be used for equity of resource allocations<br>• Using the literature, it is usually used to obtain a better geographical representation	
Evaluation of the impact level of each of these logistical aspects on the sustainability and functionality of the system	• Very important point, logistic criteria must not be used independently of other criteria<br>• This will be limited using an MCDA approach subsequent to finalizing selection criteria	• Nil, however, this point must be considered when criteria will be used e.g., using an MCDA approach
Other relevant criteria could include: Is there a need for sentinel sites within the surveillance system?	• This decision should normally have been addressed prior to using the tool	• Nil
Other relevant criteria could include: standardization of the protocol	• Important point during planning of the surveillance system<br>• Should not be impacted by sentinel unit location (unless protocol cannot be carried out, and this aspect must be considered by the decision makers, but is incorporated within the logistic criteria)	• Nil: however, standardization of the protocol will be discussed in the discussion
Other relevant criteria could include: minimum threshold for risk determined by incidence rate or abundance of ticks	• Very interesting idea and relevant to the surveillance context	• Although difficult to incorporate this idea with the decision tool, could include minimum threshold for each of the criteria during MCDA approach or general use of retained criteria; this should be discussed
May not be useful in all VBD contexts	• This is an aspect which was difficult to develop for the decision tool<br>• Realist-type review covered all types of VBDs<br>• Will remain a limitation: must be used and evaluated using different systems	• Review of all material within realist-type review<br>• However, will likely remain a limitation; must be discussed
Overall, format of the decision tool is confusing and suboptimal	• This was pointed out by several reviewers<br>• Case example is very important for illustrating the functionality of the decision tool	• Important effort was made to reduce complexity of the decision tool and make more user-friendly

How to use the criteria retained in the decision tool is not clear	• This was pointed out by several reviewers<br>• This point will be discussed within the current paper, however, will be detailed further in future work	• Nil: this point will be discussed within the current paper, however, will be detailed further in future work

9. Improvement of the decision tool

After analyzing the comments from expert reviewers and returning iteratively within the realist-type review material, an improved version of the decision tool was produced (Figure 2).

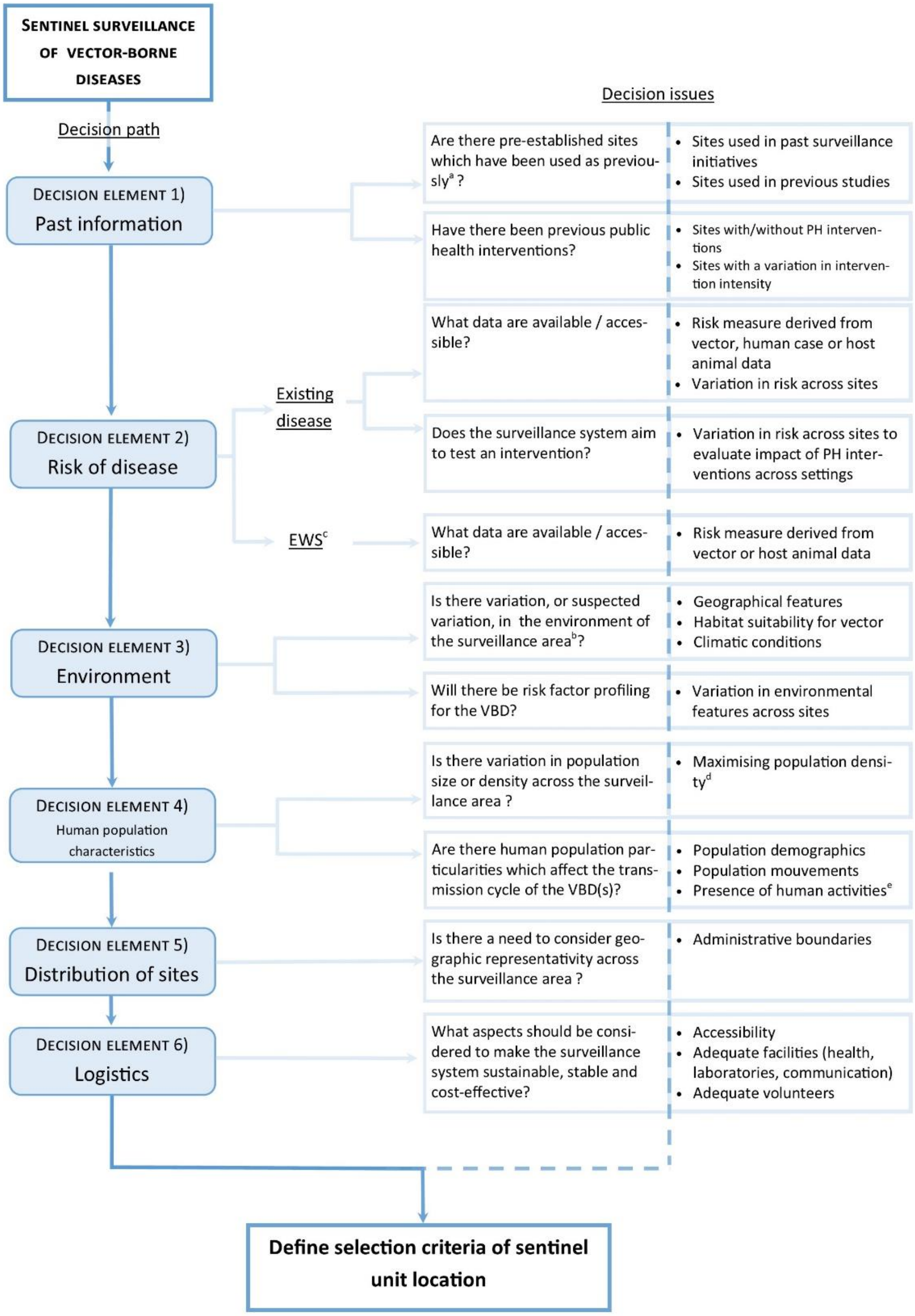

Figure 2. Decision tool for determining key criteria in developing a protocol for the selection of sentinel unit locations for vector-borne diseases

[a] Site should have been used for a similar objective
[b] The variation in the environment is judged significant by the investigators
[c] Early warning system
[d] It is also relevant to consider potential important population influx e.g., from tourism, occupational reasons
[e] Human activities which influence exposure to vectors / vector-borne diseases
